# Germline INDELs and CNVs in a cohort of colorectal cancer patients: their characteristics, associations with relapse‐free survival time, and potential time‐varying effects on the risk of relapse

**DOI:** 10.1002/cam4.1074

**Published:** 2017-05-23

**Authors:** Salem Werdyani, Yajun Yu, Georgia Skardasi, Jingxiong Xu, Konstantin Shestopaloff, Wei Xu, Elizabeth Dicks, Jane Green, Patrick Parfrey, Yildiz E. Yilmaz, Sevtap Savas

**Affiliations:** ^1^ Discipline of GeneticsFaculty of MedicineMemorial UniversitySt. John'sNewfoundland and LabradorCanada; ^2^ Department of BiostatisticsPrincess Margaret HospitalUniversity of TorontoTorontoOntarioCanada; ^3^ Dalla Lana School of Public HealthUniversity of TorontoTorontoOntarioCanada; ^4^ Clinical Epidemiology UnitFaculty of MedicineMemorial UniversitySt. John'sNewfoundland and LabradorCanada; ^5^ Discipline of OncologyFaculty of MedicineMemorial UniversitySt. John'sNewfoundland and LabradorCanada; ^6^ Department of Mathematics and StatisticsFaculty of ScienceMemorial UniversitySt. John'sNewfoundland and LabradorCanada

**Keywords:** CNVs, colorectal cancer, early‐relapse markers, genetic variation, markers with time‐varying effects, relapse‐free survival

## Abstract

INDELs and CNVs are structural variations that may play roles in cancer susceptibility and patient outcomes. Our objectives were a) to computationally detect and examine the genome‐wide INDEL/CNV profiles in a cohort of colorectal cancer patients, and b) to examine the associations of frequent INDELs/CNVs with relapse‐free survival time. We also identified unique variants in 13 Familial Colorectal Cancer Type X (FCCX) cases. The study cohort consisted of 495 colorectal cancer patients. QuantiSNP and PennCNV algorithms were utilized to predict the INDELs/CNVs using genome‐wide signal intensity data. Duplex PCR was used to validate predictions for 10 variants. Multivariable Cox regression models were used to test the associations of 106 common variants with relapse‐free survival time. Score test and the multivariable Cox proportional hazards models with time‐varying coefficients were applied to identify the variants with time‐varying effects on the relapse‐free survival time. A total of 3486 distinct INDELs/CNVs were identified in the patient cohort. The majority of these variants were rare (83%) and deletion variants (81%). The results of the computational predictions and duplex PCR results were highly concordant (93–100%). We identified four promising variants significantly associated with relapse‐free survival time (*P* < 0.05) in the multivariable Cox proportional hazards regression models after adjustment for clinical factors. More importantly, two additional variants were identified to have time‐varying effects on the risk of relapse. Finally, 58 rare variants were identified unique to the FCCX cases; none of them were detected in more than one patient. This is one of the first genome‐wide analyses that identified the germline INDEL/CNV profiles in colorectal cancer patients. Our analyses identified novel variants and genes that can biologically affect the risk of relapse in colorectal cancer patients. Additionally, for the first time, we identified germline variants that can potentially be early‐relapse markers in colorectal cancer.

## Introduction

Colorectal cancer is the third most commonly diagnosed cancer and the fourth leading cause of cancer‐related deaths worldwide 1. Both the incidence and mortality rates of this disease show variability around the world; the incidence rates are higher in developed countries, such as Japan, Australia/New Zealand, USA, Europe, and Canada 2, 3. Despite a higher rate of incidence, interestingly, the survival rates are generally much better in the developed countries compared to developing countries. For example, the 5‐year survival rate of colorectal cancer patients is around 65% in the USA and Canada, which is higher than the survival rates in developing countries 3, 4. The root cause of this geographic disparity is unknown, but variable lifestyle, socioeconomic, or environmental factors, or widespread screening and diagnostic programs in developed countries compared to the developing countries are suspected factors 2, 3. In addition to these factors, genetic factors may also influence the risk of susceptibility and disease outcomes in patients. The promise of the *personalized medicine* is that such genetic factors influencing the susceptibility may be used for prevention and screening purposes, while those predicting the prognosis may be used to predict the potential course of the disease, and thus, to inform the treatment decisions 5, 6.

Among the genetic factors are the structural variants, such as insertion/deletion (INDEL) and copy number variation (CNV) polymorphisms 7, 8. Both INDELs and CNVs are DNA segments that present at variable copy numbers (i.e., caused by deletions or insertions/amplifications) among the individuals of a population. Both types of variants can also be inherited or formed de novo. Yet, the main difference between the INDELs and CNVs is their sizes: while there is no consensus, usually those variants shorter than 1 kb are called INDELs, whereas larger variants are called CNVs. Compared to single‐nucleotide polymorphisms (SNPs), the most common type of genetic variation in the human genome, structural variations (with the exception of 1 bp INDELs) affect more nucleotides 7 and are characterized by a higher per‐locus mutation rate, and thus these variants are considered to be a major source of genetic as well as phenotypic variability in humans 8, 9. A significant portion of INDEL/CNV sequences also contain parts or the entire sequences of genes (i.e., genic INDELs/CNVs), and hence may affect gene function or expression 7, 8. Understandably, such biological effects may lead to alteration of human physiological functions, which may contribute to the pathogenesis or progression of human diseases. In fact, an increasing number of studies have shown the associations or roles of INDELs/CNVs in both Mendelian and complex diseases, including cancer 10, 11, 12.

In colorectal cancer, a small number of studies examined the germline (i.e., nontumor DNA) INDELs/CNVs and their links to disease susceptibility, including hereditary colon cancer syndromes such as Familial Colorectal Cancer Type X (FCCX) 13, 14, 15, 16. A number of studies also looked at the associations of deletion of select genes (such as *GSTM1*,* GSTT1*) with the disease outcome 17, 18, 19. However, a comprehensive identification of INDELs/CNVs in a large patient cohort and their examination in relation to survival outcomes have not been done before. In this study, we aimed to detect the germline INDEL/CNV profiles in a colorectal cancer patient cohort and to test the possible associations of common and genic INDELs/CNVs with the patient relapse‐free survival times. We also identified the rare INDELs/CNVs that are only detected in patients diagnosed with FCCX.

## Materials and Methods

### Ethics approval

This study was approved by the Health Research Ethics Authority (HREA) of Newfoundland and Labrador (Reference numbers 09.106, 13.073 and 15.294).

### Patient cohort and the genome‐wide data

The patient cohort examined in this study was previously described 20. In short, it included 505 patients out of 750, who were recruited to the Newfoundland Colorectal Cancer Registry (NFCCR) between January 1999 and December 2003 21, 22. A written consent and permission to access tissues and medical reports were obtained from patients or their close relatives. Peripheral blood samples were collected from most of the patients at the time of recruitment and were used to extract genomic DNA. Patient follow‐up was performed as described by Negandhi and his coauthors 18. Among 750, 539 stage I–IV patients with available clinicopathological and outcome data as well as germline (i.e., blood‐extracted) DNA samples were genotyped (service provider: Centrillion^®^ Biosciences, CA) using the Illumina^®^ Human Omni1_Quad_v1 genome‐wide SNP genotyping platform, as reported previously 20. This high‐resolution Illumina Infinium^®^ BeadChip is designed to provide the genome‐wide SNP genotype, as well as the signal intensity data for 1,140,419 probes (http://www.illumina.com/documents/products/datasheets/datasheet_humanomni1_quad.pdf). In this study, the signal intensity data for each patient were used as input for detection of their INDELs/CNVs. Probe locations in this platform were based on the human genome coordinate 19 (hg19), which was used throughout this project.

Subsequent to the SNP genotyping reaction of 539 patients, a set of quality control and population structure analyses was carried out as reported earlier 20. At the end, 505 Caucasian and unrelated patients constituted the initial, starting cohort in this study.

### Detection of INDELs/CNVs

**Figure 1:**
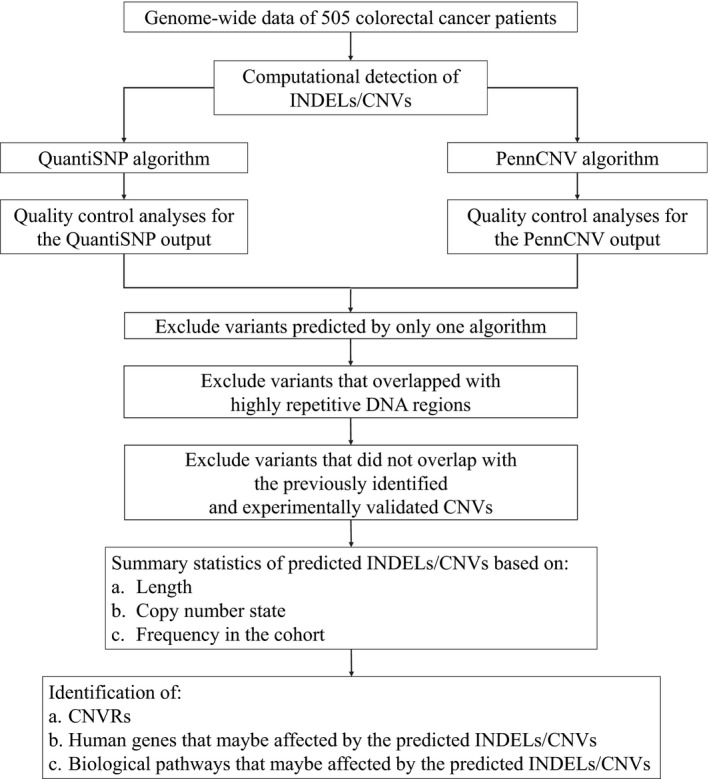
The main steps of the computational analysis that were used to detect, describe, and examine the INDELs/CNVs in the patient cohort.CNV, copy number variation; CNVR, CNV region; INDEL, insertion/deletion.

The main steps used to detect INDELs/CNVs in this study are summarized in Figure**** 1. Variants were detected using two different algorithms, QuantiSNP 23 and PennCNV 24, followed by a series of quality control and exclusion criteria as described in detail in Data S1. A total of 495 patients out of the initial set of 505 patients had satisfied these criteria, and thus, formed the final study cohort (Table 1).

**Table 1:** The baseline features of the patient cohort

Features	Number	%
Sex		
Female	194	39.19
Male	301	60.81
Age at diagnosis		
<65	312	63.03
≥65	183	36.97
Location		
Colon	328	66.26
Rectum	167	33.74
Histology		
Nonmucinous	438	88.48
Mucinous	57	11.52
Stage		
I	89	17.98
II	193	38.99
III	164	33.13
IV	49	9.90
Grade		
Well/moderately differentiated	457	92.32
Poorly differentiated	34	6.87
Unknown	4	0.81
Vascular invasion		
Absent	300	60.61
Present	158	31.92
Unknown	37	7.47
Lymphatic invasion		
Absent	290	58.59
Present	166	33.54
Unknown	39	7.88
MSI status		
MSI‐L/MSS	421	85.05
MSI‐H	53	10.71
Unknown	21	4.24
Tumor *BRAF* Val600Glu mutation		
Absent	402	81.21
Present	47	9.49
Unknown	46	9.29

MSI‐H, microsatellite instability‐high; MSI‐L, microsatellite instability‐low, MSS, microsatellite stable.

### Identification of genes and biological pathways possibly affected by the INDELs/CNVs

To identify the genes that are possibly affected by the INDELs/CNVs, an overlap (≥1 bp) analysis was performed between the distinct INDELs/CNVs and the list of expressed sequences based on the hg19 that was obtained from the ENSEMBL database on August 2014 25. These INDELs and CNVs are called as “genic INDELs and CNVs” throughout this study. In order to obtain the protein pathway information, the list of genes that overlapped with the INDELs/CNVs was loaded into the “Gene List Analysis” tool of the PANTHER database 26 on September 2015.

### Experimental validation of select INDELs/CNVs

#### Selection of CNVs

For DNA analysis, we prioritized those INDELs/CNVs that were homozygously deleted in at least 5% of the patients. Whenever possible, we aimed to further prioritize INDELs/CNVs that overlap/delete the sequence of an entire gene over those that partially overlap with genes. A literature search was also performed and functional relevance to cancer was also considered. At the end, 10 INDELs/CNVs that affect the sequences of *ADAM3A/ADAM5A*,* CNOT1*,* DLEU1*,* FAM149A*,* FILIP1L/CMSS1*,* LCE3C/LCE3B*,* NME7*,* REV1*,* WDR34/VTI1BP4*, and *WWOX* genes were selected for experimental validation.

#### Duplex end‐point PCR

Duplex end‐point PCR was performed for selected genic INDELs/CNVs in the DNA samples of 100 of the patients. This analysis can distinguish between the patients with homozygous deletion and those with at least one copy of the variant. We opted for duplex PCR rather than quantitative methods due to availability of low amount of patient DNA samples. Oligonucleotides and amplification conditions are described in Data S2.

### Statistical analyses

All statistical analyses were performed by R (version 3.2.4) 27 or SPSS (IBM‐SPSS versions 22 and 23).

#### INDELs/CNVs

The 106 variants (31 INDELs and 75 CNVs) with the following features were selected for survival analyses: (1) INDELs/CNVs whose sequences overlap with genes (i.e., genic INDELs/CNVs), and (2) INDELs/CNVs that had at least 10% (while also not exceeding 90%) of the patients with the copy number state (CN) of 0. Our hypothesis was that patients who were homozygously deleted for the CNV/INDEL sequence (and thus likely have both copies of the gene affected; CN = 0) had different survival outcomes than those patients who had at least one copy of the INDELs/CNVs (and thus with at least one copy of the gene unaffected by the INDELs/CNVs; CN ≥ 1). Hence, during the statistical analyses, patients were categorized as CN = 0 versus CN ≥ 1, where the latter group of patients served as the reference group. Information related to these CNVs/INDELs and genes are shown in Data S3.

#### Survival outcome

Relapse‐free survival (RFS) was defined as the time from diagnosis till the time of diagnosis of local or distant recurrence (i.e., metastasis), or death (whichever occurred earlier). Patients who did not experience these events were censored at the time of their last contact. For two out of 495 patients, either the relapse status or the relapse/last contact date was missing. During the entire follow‐up period, a total of 197/493 = 40% of the patients have experienced relapse.

#### Baseline variables and survival analyses

Potential multicollinearity among the baseline variables was checked using the Pearson's correlation test in R. As a result, vascular and lymphatic invasion were found to be highly correlated with each other (*r* ^*2*^ = 0.96); between the two, the one with the smaller number of missing values (i.e., vascular invasion) was included into the baseline modeling.

Survival analyses were done using the survival package in R 28. We first tested the associations of variables with RFS assuming all variables satisfied the proportional hazards (PH) assumption of the Cox PH regression model. We also tested the PH assumption for each variable and, when appropriate, modeled survival outcome using the Cox regression model with time‐varying coefficients.

##### i) Survival analysis assuming all variables satisfied the PH assumption of the Cox PH regression model

Univariable Cox PH regression model was fitted for each baseline variable; those that had a *P* < 0.1 were then analyzed in a multivariable Cox PH regression model (stage, location, sex, vascular invasion, and microsatellite instability [MSI]). Variables that remained significant in this model were disease stage, tumor location, and MSI status. We confirmed the independent associations of these variables (stage, MSI, and tumor location) with RFS in a separate model that only contained these variables. Genotypes of each INDEL/CNV were then adjusted for these baseline variables in Cox PH regression models using the coxph function in R (Data S4 **–**Table**** 1).

##### ii) Testing the PH assumption for each variable and, when appropriate, modeling survival outcome using the Cox regression model with time‐varying coefficients

We used the score test 29 to check whether the study variables violated the PH assumption (i.e., the hazard ratio does not remain constant suggesting that the effect of the variable on the RFS changes over time). Among the baseline variables in Table**** 1, age at diagnosis (defined as < 65 years of age vs. ≥ 65 years of age) was the only one that violated this assumption. Thus, we first examined the baseline variables that had a *P* < 0.1 in the univariable analyses (stage, sex, vascular invasion, location, and MSI) in an age‐stratified Cox PH regression model. As a result, disease stage, tumor location, and MSI status remained significant. Thus, the final baseline model consisted of age as stratum and disease stage, MSI status, and tumor location as variables for adjustment. Associations of each of the 106 INDELs/CNVs with RFS were then examined in these models with or without time‐varying coefficients as appropriate. To do so, we first examined each of the variants using the score test 29 under the stratified multivariable models to evaluate whether they violated or satisfied the PH assumption. Variants that satisfied the PH assumption were investigated in age‐stratified conventional Cox PH regression models (without the time‐varying coefficients) (Data S4 **–**Table**** 2). For those variants that violated the PH assumption (i.e., potential variants with time‐varying effects; score test *P *<* *0.05), we first estimated the time‐point before and after which their effects on the RFS changed by following the approach described by Pavelitz and others 30. In brief, we considered each of the time‐points (and used the survSplit and cox.zph functions in R) starting with *t*_1 _= 0.1 with 0.1 year increments till the end of follow‐up time (10.8 years) in age‐stratified multivariable models. The time‐point at which the model had the largest maximized log partial likelihood was deemed to be the time‐point where the effect of the variants on RFS changed 30. Score test was again applied to check the PH assumption before and after the identified time‐point for each variant and the coxph function was used to estimate the hazard ratios and confidence intervals for these time periods.

**Table 2:** The main features of the distinct, high‐confidence INDELs/CNVs identified in the study cohort

Variable	Number	
Total number of distinct INDELs/CNVs	3486	
Mean distinct INDEL/CNV length	35,187 bps	
Length	Number	%
INDELs	360	10.33
CNVs	3126	89.67
Frequency	Number	%
Rare INDELs/CNVs (< 5% of the patients)	2891	82.93
Common INDELs/CNVs (≥ 5% of the patients)	595	17.07
Number of INDELs/CNVs per CN state^a^	Number	%
INDELs/CNVs with two CN states	**2905**	**83.33**
(CN = 0) Two copy deletion	685	19.65
(CN = 1) One copy deletion	1596	45.78
(CN = 3) One copy duplication	607	17.41
(CN = 4) Two or more copy duplication	17	0.49
INDELs/CNVs with multiple CN states	**581**	**16.67**
A. INDELs/CNVs with three CN states	**577**	**16.55**
CN = 0 or 1	543	15.58
CN = 0 or 3	7	0.20
CN = 0 or 4	2	0.06
CN = 1 or 3	13	0.37
CN = 3 or 4	12	0.34
B. INDELs/CNVs with four CN states	**4**	**0.12**
CN = 0, 3 or 4	1	0.03
CN = 0, 1 or 4	1	0.03
CN = 0, 1 or 3	2	0.06

CN, Copy number state; CNV, copy number variation; INDEL, insertion/deletion.

a The “normal” CN state of 2 copies is not shown.

A *P* < 0.05 was assumed significant. Because of the exploratory nature of this study and in order to limit false‐negative results, a correction for multiple testing was not performed.

## Results

### Characteristics of the distinct INDELs/CNVs

Baseline characteristics of 495 patients whose data passed the quality control thresholds by both QuantiSNP and PennCNV algorithms and who constituted the final cohort of patients are summarized in Table**** 1.

**Figure 2:**
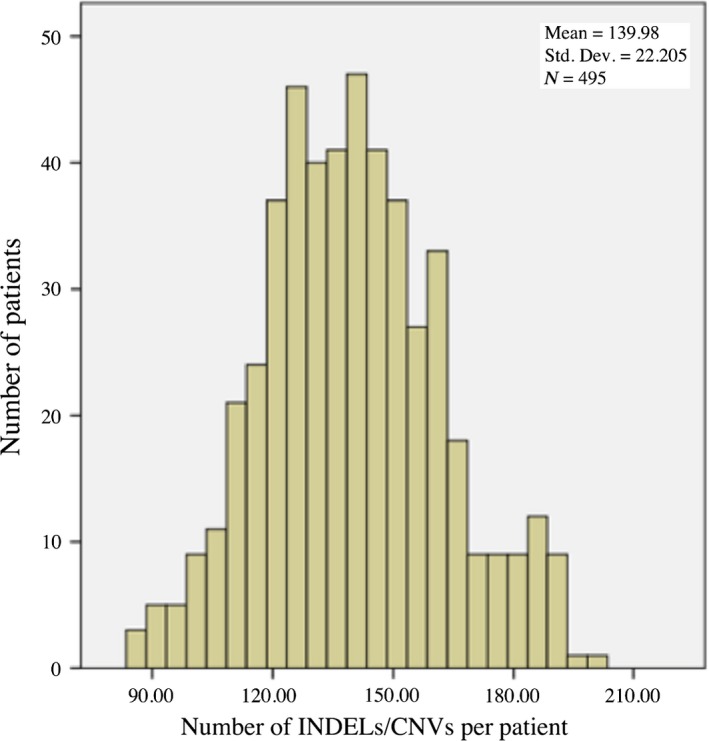
Distribution of the number of predicted INDELs/CNVs in the patient cohort. CNV, copy number variation; INDEL, insertion/deletion.

Collectively, in all patients, 3486 distinct INDELs/CNVs (Table**** 2) were identified, each of which had unique start and end positions and was detected in at least one patient. The sizes of these distinct variants ranged from 359 to 956,373 bps with a mean length of ~35 kb. The average number of distinct variants per patient was 140 (Fig.**** 2). CNVs and deletion variants constituted ~90% and 81% of the variants, respectively. About 83% of the distinct variants were rare, occurring in less than 5% of the patients, whereas ~17% of the variants were common occurring in at least 5% of the study cohort. Additionally, the majority of the variants (83.3%) had two CN state (i.e., bi‐allelic), while the rest were multi‐allelic (Table**** 2). Overall, distinct variants were located within 1527 different CNVRs.

### Genes and pathways that may be affected by the distinct INDELs/CNVs

**Figure 3:**
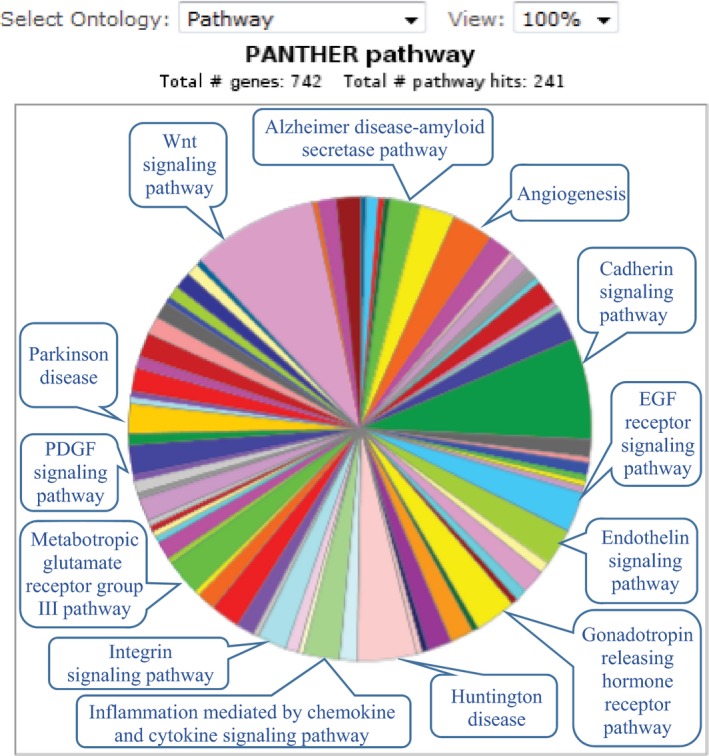
PANTHER database results showing the major biological pathways possibly affected by the INDELs/CNVs. CNV, copy number variation; INDEL, insertion/deletion.

Out of 3,486 distinct INDELs/CNVs, 2,209 (63.4%) variants overlapped with the sequences of 1673 genes (Table**** 3). The entire sequence of 793 genes overlapped with the sequence of a variant; these variants thus may change the gene dosage and affect the transcript levels. A total of 134 genes were affected by multiple INDELs/CNVs, representing possible hot‐spots. Frequencies of the INDELs/CNVs changed between 0.2% and 45.1% in the patient cohort. The PANTHER database returned information for 742 genes acting in 241 biological pathways. The main protein pathways that contained the genes affected by the variants are depicted in Figure**** 3.

**Table 3:** Genes possibly affected by the INDELs/CNVs

Affected genes	Numbers
Genes completely covered by INDELs/CNVs	659
Genes partially overlapped with INDELs/CNVs	880
Genes completely or partially overlapped with different INDELs/CNVs	134

CNV, copy number variation; INDEL, insertion/deletion.

### DNA analysis

Duplex PCR analysis showed that the results of the computational and experimental analyses agreed in 93–100% of the cases (Data S2 **‐**Table**** 1). Specifically, in the majority of the cases (*n* = 7) the concordance rates were 100%, while in three variants we obtained concordance rates of 99%, 98%, and 93%. The lowest concordance rate (93%) was observed in the case of a CNV located in a duplicated gene region (*LCE3C/LCE3B*).

### INDELs/CNVs in FCCX cases

There were 13 FCCX cases in our patient cohort. In order to explore whether there were INDELs/CNVs unique/specific to these patients, we first compared the unique and high‐confidence variant data of the 13 patients with the rest of the patients in our cohort. As a result, we have identified 28 variants in 11 FCCX patients that were unique to the FCCX cases (Data S5). Twenty‐one of these variants affected at least one gene and none of the CNVs or the genes were detected in more than one patient. However, there were two patients who had different variants at chromosome 6p22.1 that overlapped with each other (Data S5 **‐**Table**** 1). Second, considering the possibility that rare variants that may be specific to FCCX cases could have been eliminated during the quality control analyses (particularly when we have filtered out the variants that were not detected in previous studies 31, 32, 33), we also looked at the variant data of FCCX cases eliminated at this stage. As a result, there were 30 variants (25 affecting at least one gene) in 13 FCCX cases, which were not identified in other patients in our cohort or the individuals in three other previous studies (Data S5 **–**Table**** 2).

### Examination of INDELs/CNVs in relation to relapse‐free survival of patients

Assuming that the PH assumption held for all variables, our results showed that two CNVs (located within the introns of *TGFBR3*, and *STEAP2‐AS1* and *STEAP2* genes) and one INDEL (located within the intron sequences of the *CMSS1* and *FILIP1L* genes) were associated with the relapse‐free survival time when adjusted for prognostic factors (Data S4 **–**Table**** 1). In the case of the *CMSS1* and *FILIP1L* INDEL, patients with homozygous deletion had increased risk of relapse compared to patients with at least one copy, whereas those patients having homozygous deletion of the *TGFBR3* or *STEAP2‐AS1* and *STEAP2* CNV sequences had reduced risk of relapse compared to patients who had no homozygous deletion of these variants.

We then checked the PH assumption starting with the baseline variables and found that age at diagnosis had time‐varying effect on RFS; patients who were younger than 65 were at significantly increased risk of recurrence, metastasis, or death in the initial 2.1 years relatively to the patients who were 65 or older at the time of diagnosis, whereas after this time period, the direction of the effect was reversed (i.e., HR: 0.44, *P *=* *0.006 and HR: 1.6, *P *=* *0.0075, respectively). Thus, we reanalyzed the associations of the variants in age‐stratified multivariable models. These analyses identified three variants that have potential time‐varying effects on relapse‐free survival (Table**** 4). Associations of two of these variants with the relapse‐free survival time remained significant prior to their time‐points where the effect on the relapse‐free survival changed (around 3 years postdiagnosis; Table**** 4). These CNVs were located within the *PDLIM3* and *GUSBP1* genes and patients with the homozygous deletions of these CNVs had increased and decreased risk of relapse during the initial years after diagnosis, respectively. In the case of the remaining 103 variants that satisfied the PH assumption, in addition to *TGFBR3*,* STEAP2‐AS1* and *STEAP2*, and *CMSS1* and *FILIP1L* variants, association of a new variant overlapping with the sequence of the *RP11‐143P4.2* gene was detected in age‐stratified models (Table**** 5; Data S4 **–**Table**** 2). All of these CNVs/INDELs were located within the intron sequences of the genes.

**Table 4:** Results of the Cox regression models with time‐varying coefficients for the three variants that violated the proportionality assumption

Time‐point (years)	Variables in the model	HR	95% CI for HR (lower)	95% CI for HR (higher)	*P*‐value	*P* ‐value for PH assumption test
4.3	Stage (II vs. I)	1.433	0.856	2.398	0.171	0.588
	Stage (III vs. I)	2.266	1.374	3.736	**0.001**	0.568
	Stage (IV vs. I)	5.950	3.441	10.289	**1.74E‐10**	0.146
	Location (Rectum vs. colon)	1.411	1.046	1.904	**0.024**	0.111
	MSI status (MSI‐H vs. MSS/MSI‐L)	0.327	0.152	0.708	**0.005**	0.230
	aChr1_169207360_169241309 (0 CN vs. 1 or 2 CN) (*NME7*)					
	Before the time‐point	1.400	0.848	2.310	0.188	0.906
	After the time‐point	0.159	0.022	1.153	0.069	0.898
2.6	Stage (II vs. I)	1.502	0.899	2.509	0.120	0.832
	Stage (III vs. I)	2.390	1.450	3.940	**0.001**	0.800
	Stage (IV vs. I)	6.591	3.807	11.412	**1.65E‐11**	0.082
	Location (Rectum vs. colon)	1.419	1.051	1.916	**0.022**	0.183
	MSI status (MSI‐H vs. MSS/MSI‐L)	0.315	0.145	0.683	**0.003**	0.206
	aChr4_186441932_186444110 (0 CN vs. 2 CN) (*PDLIM3*)					
	Before the time‐point	2.108	1.317	3.373	**0.002**	0.794
	After the time‐point	0.726	0.423	1.245	0.244	0.864
2.8	Stage (II vs. I)	1.477	0.883	2.470	0.138	0.678
	Stage (III vs. I)	2.354	1.428	3.879	**0.001**	0.693
	Stage (IV vs. I)	5.952	3.448	10.274	**1.52E‐10**	0.086
	Location (Rectum vs. colon)	1.421	1.052	1.919	**0.022**	0.103
	MSI status (MSI‐H vs. MSS/MSI‐L)	0.323	0.149	0.700	**0.004**	0.224
	aChr5_21450792_21452439 (0 CN vs. 2 CN) (*GUSBP1*)					
	Before the time‐point	0.416	0.182	0.955	**0.039**	0.770
	After the time‐point	1.511	0.927	2.463	0.098	0.848

Chr, chromosome; CI, confidence interval; CN, copy number state; HR, hazard ratio; MSI‐H, microsatellite instability‐high; MSI‐L, microsatellite instability‐low; MSS, microsatellite stable; PH, proportional hazards; *P* < 0.05 are bolded.

a Genes that overlap with the variants are shown in parentheses.

**Table 5:** Variants that satisfied the proportionality assumption and significantly associated with the relapse‐free survival time

Gene	Variant	*P* ‐value	HR	95% CI (lower)	95% CI (higher)
*TGFBR3*	Chr_1_92232111_92233227 (0 CN vs. 2 CN)	0.0454	0.5211	0.2752	0.9867
*CMSS1, FILIP1L*	Chr_3_99628822_99629567 (0 CN vs. 1 or 2 CN)	0.015	1.6936	1.1076	2.5896
*RP11‐143P4.2*	Chr_3_192875738_192885153 (0 CN vs. 2 or 4 CN)	0.0394	1.3586	1.0149	1.8186
*STEAP2‐AS1, STEAP2*	Chr_7_89810608_89812114 (0 CN vs. 2 CN)	0.0372	0.5776	0.3447	0.968

Chr, chromosome; CI, confidence interval; CN, copy number state; HR, hazard ratio.

## Discussion

In this study, we detected the genome‐wide INDEL/CNV profiles of 495 Caucasian colorectal cancer patients from Newfoundland, Canada, using two CNV detecting algorithms and stringent quality control measures. Further analyses were performed to test the associations of 106 genic and common variants with the patient outcomes. The potential time‐varying effects of these variants on relapse‐free survival times were also investigated. Additionally, we explored the rare and unique INDELs/CNVs that are only observed in 13 hereditary colon cancer syndrome patients diagnosed with FCCX.

Our results showed that, similar to other studies, QuantiSNP and PennCNV detected different numbers of variants in the patient genomes, which can be attributed to the different methodologies applied by these algorithms 34, 35. However, when a variant was detected by both algorithms, the genomic positions and borders of the variants were identical in the majority of the cases (84.3%), suggesting a high concordance rate for variants detected by both QuantiSNP and PennCNV. In addition, 97% of the variants after the quality control measures had at least 50% of their sequences overlap with the variants previously identified by other groups. These results are in agreement with others' findings 34, 35, 36 that the false‐prediction rate decreases when multiple algorithms and strict quality control measures are used for INDEL/CNV detection. This was further supported by the DNA analysis of 10 of the variants in our study, which showed a fairly high concordance rate between the DNA analyses and the computational predictions.

The majority of the variants identified in this study were deletions (Table**** 2). This is expected as when a genome‐wide signal intensity data are used, deletion variants are detected easier than duplication variants (CN ≥ 3) 24. Also, our list of variants contains mostly the large variants (i.e., CNVs with sizes of at least 1 kb). This too is expected because the QC measures inclined toward removing smaller variants. For example, during this study, variants with sizes <10 bps or detected by <10 probes were eliminated from the variant calls to remove the potential false‐positives. These criteria inevitably should have resulted in exclusion of a portion of the short variants. Of note, the shortest high‐confidence variant identified in our study had a length of 359 bps. Therefore, while it is likely that our variant data are missing a portion of variants due to the strict QC measures, our QC measures also served to reduce the false‐positive predictions, increased the accuracy of our results, and at the end yielded INDELs/CNVs that are deemed to be detected with high confidence.

The sequences of a number of variants we identified overlap with the human gene sequences. These “genic” INDELs/CNVs are biologically interesting as they can delete or duplicate gene sequences, and as a result may affect physiological functions. Overall, our data showed that the number of gene sequences affected by rare variants (*n* = 1538) were higher than the number of gene sequences affected by common variants (*n* = 135). Similar to others' findings, these results may be explained by the fact that variants that affect genes are kept at low frequencies in the populations 37. Additionally, the genes that harbor INDEL/CNV sequences come from a variety of biological pathways (Fig.**** 3), some of which are established in cancer development or progression; notably WNT signaling and angiogenesis pathways 38, 39, 40, 41. Variants identified in this study hence deserve further investigation as it is possible that some of them are biologically linked to susceptibility or prognosis in colorectal cancer.

Considering that rare INDELs/CNVs may lead to high‐penetrant genetic disorders including FCCX, as part of this study, we also explored the variant data in 13 FCCX cases. FCCX is a familial colon cancer syndrome where patients satisfy the clinical criteria for hereditary nonpolyposis colorectal cancer (HNPCC) but have tumors that lack the microsatellite instability 42. Many different genetic approaches including linkage, association, CNV, and mutation screening studies, have been performed in FCCX cases/families. While these studies have identified several candidate genes and genetic regions, the entire body of findings suggests genetic heterogeneity and lack of a common genetic cause among unrelated FCCX cases 14, 43, 44, 45. In this study, we have examined the INDEL/CNV profiles of the FCCX cases in our cohort and identified a number of rare variants that were unique to the FCCX patients. Our results, however, did not identify a gene or INDEL/CNV that was detected in multiple unrelated cases (although we have identified two patients with overlapping variants on chromosome 6p22.1). Thus, our data largely agree with previous findings and do not provide an evidence of specific rare variants or genes that can explain this disease in more than one FCCX patients. We also compared our findings with the others in the literature. A study by Masson et al. 14 suggested the involvement of CNVs, at least to some extent, in FCCX development. A comparison of the INDELs/CNVs only detected in our FCCX patients (Data S5) and Masson's group did not identify a common variant or gene affected by the variants in our list. However, there were a number of CNVs/INDELs in our data that were located within or around the genomic regions previously identified in linkage analyses (summarized in Sanchez‐Tome et al. 2015; 45). These INDELs/CNVs thus may form an interesting list of candidate variants for further studies that can dissect the potential INDEL/CNV – FCCX relationship.

Considering the fact that colorectal cancer patients have increased risk of death as well as recurrence and metastasis after their initial diagnosis/treatment 3, 4, 46, we also examined the associations of baseline clinical factors and 106 CNVs/INDELs with the survival outcome in our patient cohort. We note that while the results obtained are generally quite similar, since it is the proper model for variants that violate the proportionality assumption, we consider the results of the Cox regression model with time‐varying coefficients (Table**** 4) more accurate than the results of the conventional Cox PH regression model. One of the interesting findings of this analysis was that the hazards ratio of age at diagnosis categories (<65 years vs. ≥ 65 years) changed over time. Specifically, relatively young age at diagnosis (< 65 years) was associated with increased risk of relapse within the first ~2 years after diagnosis, while after this initial time period the risk of relapse increased for the older patients (≥ 65 years). The exact reason of this time‐varying effect in our patient cohort is not known, but it can be linked to aggressive or advanced disease at diagnosis in relatively younger patients in our cohort (46.8% stage III and IV patients in <65 years of age category compared to 36.6% stage III and IV patients in the ≥65 years of age category). Although different criteria are used for young patient classification in other studies (which is usually <40 years of age 47, 48, 49, 50), this is consistent with the other published reports where the younger patients were reported to be more likely to be diagnosed at later stages and have increased chance of recurrence early after diagnosis 46, 51.

As per the genetic variants, our analyses identified a total of six genic variants (five CNVs and one INDEL) that were associated with the relapse‐free survival time in the patient cohort (Tables 4 and 5). The sizes of these variants changed from 746–9416 bp and all were located in noncoding (i.e., intronic) parts of the genes. The genes that may be affected by these variants function in a variety of biological pathways; *PDLIM3* codes for a cytoskeletal protein; *GUSBP1* codes for an expressed pseudogene with unknown functions; *TGFBR3* codes for a TGFβ signaling pathway protein; *STEAP2‐AS1* codes for the antisense RNA for *STEAP2*, and *STEAP2* codes for a transmembrane metalloreductase; *RP11‐143P4.2* codes for a long noncoding RNA; and *CMSS1* codes for a ribosomal small subunit homolog and *FILIP1L* codes for a filamin A‐binding‐like protein. Some of these genes were previously linked to carcinogenesis and disease progression. For example, *TGFBR3* is a potential tumor suppressor gene deleted in various cancers and with a role also in cell migration, invasion, and metastasis 52. Interestingly, one study reported its expression being associated with reduced apoptosis and increased migration in a colon cancer cell line 53. Additionally, FILIP1L has been shown to have a role in inhibition of WNT signaling pathway, a pathway implicated in colorectal cancer and metastasis 37, 38 as well as in cellular invasion in an ovarian cancer model 54 and colon cancer cell lines 55. Consistent with these results, another study showed that reduced levels of this protein in colorectal tumors were associated with reduced overall survival times of patients 56. While it is currently unknown whether these INDELs/CNVs have biological effects on the corresponding genes (and hence, have direct effects on the disease progression and risk of relapse in colorectal cancer), it is quite possible as a large number of noncoding sequences in the human genome contain regulatory elements 57.

Literature search showed that none of these six variants were previously linked to outcome in colorectal cancer patients, or patients diagnosed with other cancers. Interestingly, we identified that the relationships of two of these variants with the risk of relapse have varied with time (Table 4). Specifically, the hazard ratios by the *GUSBP1* and *PDLIM3* CNVs fluctuated over time, with a statistically significant associations detected only early after diagnosis (i.e., within the first ~3 years), but not after these years. Both of these CNVs are common variants presenting in 14% and 20% of the patient cohort (*GUSBP1* and *PDLIM3* CNVs, respectively). These results may be explained by these genetic variants either directly and biologically affecting the risk of recurrence/metastasis, or death, or being correlated with a yet unknown factor(s) that modifies the risk of relapse during this time period. We also note that their associations were detected only when the statistical analyses considered the time‐varying effects; otherwise these associations were missed when conventional Cox regression method was used (Data S4). This highlights the importance of using appropriate statistical approaches that can help uncover novel findings that are otherwise prone to be missed. Currently, examining the potential time‐varying effects of genetic polymorphisms/mutations on the risk of outcome is quite a rare practice. To the best of our knowledge, previously only one study has examined and identified a genetic marker with a possible time‐varying effect on the risk of outcome in colorectal cancer. In short, Pavelitz et al. 30 examined the *MRE11* gene mutation status in stage III colorectal cancer patients and found that the proportionality assumption of the Cox modeling was violated for overall and disease‐free survival times in their patient cohort. These authors then moved on with a statistical approach that we adopted in our analysis, including identification of a time‐point and modeling survival outcome using the Cox regression model with time‐varying coefficients 58. Therefore, the mutant *MRE11* these authors identified and the germline *GUSBP1* and *PDLIM3* CNVs our study identified are the first examples of genetic markers that potentially have time‐varying effects on patient outcomes in colorectal cancer. Overall, we conclude that the *GUSBP1* and *PDLIM3* CNVs are potential early‐relapse markers in colorectal cancer, and if results obtained in this study are replicated they can be useful not only in developing more informative prognostic models but also in elucidating the biological basis of variable risk of relapse (i.e., risk of recurrence, metastasis, or death) among colorectal cancer patients.

Like other studies, this one has strengths and limitations. Our main strengths were the following; (1) the Illumina^®^ Omni‐1‐quad platform used to generate the genome‐wide signal intensity data and helped detection of INDELs/CNVs is a high‐resolution platform, which facilitates a more efficient variant detection compared to many other platforms; (2) two CNV detection algorithms and stringent quality control/filtering steps were used in order to reduce the false‐positive predictions; (3) the results of the computational INDEL/CNV detection and the duplex PCR analysis were largely concordant; (4) this is the first large‐scale analysis of germline genic INDELs/CNVs and their relation to relapse‐free survival in colorectal cancer; (5) this is the first study that identified germline polymorphisms with time‐varying effects on patient outcome in colorectal cancer; and (6) the patient cohort was a well‐described cohort with a long follow‐up time, which increased our study power. Our limitations were; (1) variants from sex chromosomes were not included in the computational analyses; (2) while our approach detected INDELs, a significant portion of the INDELs remained unidentified as the detection parameters were geared toward detection of larger variants; (3) rare variants were not examined in relation to survival outcomes; (4) the experimental analyses were limited to duplex PCR assessing the homozygous deletion and copy number states ≥ 1 rather than quantitative techniques that could detect the individual copy number states; (5) the patient cohort was of Caucasian ancestry, thus the results may not be applicable to patients from other populations.

In conclusion, this is one of the first studies that identified the genome‐wide INDEL and CNV profiles in a large cohort of colorectal cancer patients. Our variant data are in line with the results of other studies reported in the literature. This is also the first study that comprehensively investigated the possible associations of genic INDELs/CNVs with relapse‐free survival time in colorectal cancer. We identified six variants that are candidate prognostic markers and should be examined in further studies. This is also the first study that examined and identified two CNVs that have time‐varying effects on clinical outcomes of colorectal cancer patients; if replicated, these CNVs can be used as early‐relapse markers during prognostication. Last but not the least, this study suggests that similar to other literature findings there was no one, unique, and rare INDEL or CNV that could explain the risk of FCCX in unrelated patients. Overall, this study has important implications for the future studies of INDELs/CNVs and susceptibility and prognosis in colorectal cancer.

## Conflict of Interest

None declared.

## Supporting information


**Data S1.** Detection of INDELs/CNVs.

**Data S2.** Oligonucleotides and amplification conditions for duplex end‐point PCR.

**Data S3.** Information on the 106 INDELs/CNVs.

**Data S4.** Summary of statistical analyses.

**Data S5.** Variants unique to FCCX cases.
