# Supplementary material for: Germline INDELs and CNVs in a cohort of colorectal cancer patients: their characteristics, associations with relapse‐free survival time, and potential time‐varying effects on the risk of relapse
Source: Cancer Med. 2017 May 23;6(6):1220–32. doi: 10.1002/cam4.1074 (PMC5463068; doi:10.1002/cam4.1074)
Supplement: Supplementary file 1 — Data S1. Detection of INDELs/CNVs. [file CAM4-6-1220-s001.docx]

**Supporting Information 1**

**Detection of INDELs/CNVs**

Since the majority of the recent CNV studies recommended using more than one CNV detection algorithm to increase the prediction accuracy and to decrease the false positive findings (1-3) we used two well-assessed and widely-used CNV calling algorithms; PennCNV (4) and QuantiSNP (5). Both of these algorithms require signal intensity files of each subject as inputs, which were created using a custom Perl program by merging two types of data files obtained during the genotyping reaction. These data files were; a) the report data files, which included signal intensity data (Log R ratio [LRR] and B allele frequency [BAF] values) obtained during the genotyping reaction for each marker, and b) the final report MAP file that included the chromosome numbers, marker names, and marker positions based on the human genome assembly hg19. The signal intensity files generated for each of the patients were then used by the CNV calling algorithms.

QuantiSNP (version 2) package was downloaded from the QuantiSNP download website (<https://sites.google.com/site/quantisnp/downloads>) on April 2013 and run using the default parameters (5). Since the differences in the GC contents among different genomic regions may lead to “genomic waviness” in the signal intensity data and complicate the detection of INDELs/CNVs (6), while running QuantiSNP a GC correction step was also performed.

The PennCNV package was downloaded on May 2013 from the PennCNV website (<http://www.openbioinformatics.org/penncnv/>). To detect the INDELs/CNVs by the PennCNV algorithm, new Population Frequency of B allele (PFB) and GC-model files were required. This was because the PFB and GC-model files provided by the PennCNV website were based on hg18 whereas our data was based on hg19.

To generate the new PFB file, an Illumina® Human Omni1_QuadV1 dataset containing the signal intensity files for 88 HapMap CEU (Caucasian) individuals was downloaded on May 2013 from the Gene Expression Omnibus (GEO) database (7) (platform number: GPL8882 and series number: GSE17197). These signal intensity files were previously created based on the hg18 genome coordinates and uploaded to the database by Illumina®. Second, the HumanOmni1-Quad v1.0 Build 36 to Build 37 Mapping Information file (also named HumanOmni1-Quad_v1-0_B-H_MappingInformation.txt), which included the hg18 genome coordinate information and their equivalent for the hg19, was downloaded on January 2014 from the Illumina® support website (<http://support.illumina.com/downloads/humanomni1-quad_product_support_files.html>). This mapping information file was then used to substitute the hg18 genome coordinate information with the hg19 information in the 88 HapMap CEU signal intensity files using custom Perl programs. Finally, the reformatted 88 HapMap CEU signal intensity files were used to generate the final PFB file using the Perl program *Compile_PFB.pl* provided within the PennCNV package (4).

In order to generate the GC-model file (required to correct for genomic waviness) based on the hg19 genome coordinates, we utilized two data files; the first one was the *GC5Base.txt* file that contained the percentage of the GC bases in 5-base windows based on the hg19 genome coordinates. This file was downloaded on January 2014 from the University of California Santa Cruz (UCSC) (8) genome bioinformatics download website (<http://hgdownload.cse.ucsc.edu/goldenpath/hg19/database/gc5Base.txt.gz>) as suggested by the PennCNV developers. Second, we chose the signal intensity file of a randomly selected patient and used it in the Perl program *Cal_gc_snp.pl* that was provided with the PennCNV package to generate the GC model file based on hg19. After the generation of the required input files (that is, patient signal intensity files, PFB file and GC model file all based on the hg19 genome coordinates), INDELs/CNVs in the patient genomes were predicted by the Perl program *Detect_cnv.pl* of PennCNV using the default parameters.

Since it has been noted earlier (4) that PennCNV tends to split large CNVs into smaller ones when SNP genotype array data are used for variant detection, the adjacent variants detected by PennCNV were merged together following the run. This step was performed using the *Clean_cnv.pl* program of the PennCNV package. During this step, variants were merged if the sequence gap between the variants did not exceed 1/2 of the total distance from the start position of the first variant to the end position of the second variant.

In both the QuantiSNP and PennCNV analyses, similar to many other studies (9,10) the INDEL/CNV predictions were limited to the autosomal chromosomes due to the hemizygosity in sex chromosomes that complicates the variant detection.

Initially, QuantiSNP and PennCNV detected 336,288 and 204,439 INDELs/CNVs in the genomes of the 505 patients, respectively (**Supporting Information 1-Table 1**). Overall, QuantiSNP detected more variations than PennCNV, both algorithms predicted a higher number of CNVs than INDELs, and deletions constituted the majority of the variants.

**Supporting Information 1-Table 1.** The main features of the INDELs/CNVs initially predicted by QuantiSNP and PennCNV.

| Number of INDELs/CNVs | | QuantiSNP | | PennCNV | |
| --- | --- | --- | --- | --- | --- |
| Total predicted INDELs/CNVs in the cohort | | 336,288 | | 204,439 | |
| Average number of INDELs/CNVs per individual | | 665.92 | | 404.83 | |
|  | | | | | |
| **Type** | | **N** | **%** | **N** | **%** |
| INDELs | | 76,854 | 22.85 | 46,616 | 22.80 |
| CNVs | | 259,434 | 77.15 | 157,823 | 77.20 |
|  | | | | | |
| **INDELs/CNVs per CN state** | | **N** | **%** | **N** | **%** |
| (CN= 0) | Two copy deletion | 76,035 | 22.61 | 57,698 | 28.22 |
| (CN= 1) | One copy deletion | 128,908 | 38.33 | 94,917 | 46.43 |
| (CN= 3) | One copy duplication | 64,217 | 19.1 | 49,983 | 24.45 |
| *(CN= 4, 5) | Two or more copy duplication | 67,128 | 19.96 | 1,841 | 0.90 |

**N**: Number, **CN**: Copy number state. *Please note that QuantiSNP (5) assigns the CN state 4 for variants that exist in 4 copies and CN state 5 for variants that exist in 5 or more copies in a genome. However, PennCNV assigns the CN state 4 for variants that exist in 4 or more copies in a genome (4).

**Quality control (QC) analysis and further characterization of INDELs/CNVs**

After the initial predictions of the variants, the QC files generated by the QuantiSNP and PennCNV for both the patient data and the variants detected were used to exclude samples and variants with low quality. QC parameters implemented were selected based on other groups’ works (**Supporting Information 1-Table 2**). Perl programming (including custom programs written for specific purposes as well as the Perl program *filter_cnv.pl* provided in the PennCNV website) was used to identify and exclude the data that do not meet the QC thresholds. After the post-detection QC analyses, a total of 85,469 INDELs/CNVs from 501 patients and 159,050 INDELs/CNVs from 497 patients satisfied the QC criteria of QuantiSNP and PennCNV, respectively. The data from 495 out of 505 patients satisfied the QC requirements of both algorithms and were used in the remaining steps of the study.

**Supporting Information 1 - Table 2.** Exclusion criteria for the subjects and INDELs/CNVs detected.

| **Exclusion Criteria** | | **QuantiSNP** | **PennCNV** | **References** |
| --- | --- | --- | --- | --- |
| **Subject filtering** | LRR Standard Deviation (LRR_SD) | > 0.28 | > 0.28 | (1,11) |
|  | BAF Standard Deviation (BAF_SD) | > 0.20 | - | (12) |
|  | BAF drift | - | > 0.01 | (11) |
|  | LRR waviness factor (WF) | - | ≤ - 0.04 and ≥ 0.04 | (13)* |
|  | BAF median | - | < 0.45 or > 0.55 | (14,15) |
|  | INDEL/CNV number per sample | > Mean + 3 SD | > Mean + 3 SD | (1,2) |
|  | Samples with extremely long CNVs | > 7.5 Mbps | > 7.5 Mbps | (1,2) |
| **INDEL/CNV filtering** | Variant length | < 10 bps | < 10 bps | (16,17) |
|  | Number of probes per INDEL/CNV | < 10 probes | < 10 probes | (18,19) |
|  | Confidence Score | < 30 (Max Log Bayes Factor) | <10 | (11,12,15,16,20-22) |

BAF: B Allele Frequency; bp: base pair; CNV: Copy Number Variation; INDEL: Insertion/Deletion; LRR: Log R Ratio; SD: Standard Deviation; WF: Waviness Factor. *http://www.openbioinformatics.org/penncnv/.

Further inclusion/exclusion filtering were performed to reduce the methodological artifacts, minimize the false positive findings, and eliminate the low quality data as follows:

**a)** Plink statistical tool (23) was utilized to check whether either of the algorithms predicted two INDELs/CNVs that were overlapping with each other in the same patient’s genome. Such variants would be predicted by mistake twice by an algorithm. Of note, we have detected such variants in neither the QuantiSNP nor the PennCNV outputs.

**b)** A custom Perl program was written to identify the INDELs/CNVs in the same patient that were predicted by both algorithms with the same copy number state (CN) and had sequences overlapping by at least 50% of their lengths. The overlapped variations (n=74,261) were assumed to be same variants that were detected by both algorithms. Interestingly, a large number of these variants (n=62,567, 84.3%) had identical start and end positions. This indicates that the high concordance of border detection if variants are detected by both PennCNV and QuantiSNP. Variations that did not satisfy these criteria were assumed to be predicted by one algorithm only and removed from the list of variants (1,3). When the borders of the overlapped variants were different from each other, they were merged together and the new variant borders were determined by the smallest start position and the largest end position of the merged variants (1).

**c)** It is a common practice to exclude INDELs/CNVs detected within the highly repetitive DNA regions, such as centromere and telomere regions, leukocyte immunoglobulin-like receptor gene cluster, and olfactory receptor (OR) gene regions that can complicate the INDEL/CNV detection (4,9). Hence, any variant that intersected at least 1 bp with these DNA regions were excluded from further analyses by Perl programming. To do so, first a list of centromere and telomere regions, leukocyte immunoglobulin-like receptor gene cluster and OR gene coordinates was generated based on hg19 following a number of resources. In short; **i)** the genome coordinate information for leukocyte immunoglobulin-like receptor gene cluster based on hg18 was obtained from the PennCNV website (<http://penncnv.openbioinformatics.org/en/latest/>) on February 2014. Then, the “LiftOver” tool of the UCSC (8) genome browser was then used on February 2014 to change the genome coordinates of the leukocyte immunoglobulin-like receptor gene cluster from hg18 to hg19 genome coordinates; **ii)** the list of centromere positions based on hg19 was obtained from the PennCNV website and were adjusted by adding and subtracting 100 kbps to upstream and downstream of each centromere, respectively, following the PennCNV recommendations; **iii)** the UCSC (8) genome browser was utilized to identify the start and end positions of each chromosome based on hg19. Then the telomere regions were determined by adding and subtracting 500 kbps at the start and end positions of each chromosome respectively, as suggested in the PennCNV package; and finally, **iv)** the hg19 genome coordinates of OR genes (n=840) were downloaded from the Human Olfactory Receptors Data Explorer (HORDE) database (<http://genome.weizmann.ac.il/horde/>) on February 2014 (24).

After excluding 2,905 INDELs/CNVs that overlapped at least 1 bp with highly repetitive genomic regions, 71,356 variants remained in the dataset.

**d)** Similar to other studies (15,25), INDELs/CNVs that overlap with the previously reported variants (i.e. variants detected by DNA analyses in other studies) were identified. This step was undertaken to further remove possible false predictions from our results and to identify the variants that were most likely to exist in patient genomes (i.e. not false-positives). The sequence overlap criterion was at least 50%. This reference variant information was based on three studies (26-28) that was available at the Database of Genomic Variants (DGV) (29). As a result, around 97% of the INDELs/CNVs (n=69,290) were found to overlap at least 50% of their lengths with the previously and experimentally identified variants. These INDELs/CNVs were thus detected with a “high confidence”, were highly likely to exist in the patient genomes, and constituted the final list of the INDELs/CNVs of this study (**Table 2**).

Within the patient cohort, all high confidence variants with the same borders were considered as the same variant and called as “distinct” variants throughout the manuscript. Also, all distinct variations overlapped at least 1 bp with each other were also clustered in copy number variations regions (CNVRs).

Throughout the study, PLINK (23) was utilized to handle and modify data/files, and to define and describe the INDELs/CNVs based on their lengths, frequency and copy number (CN) states.

**References**

(1) Pinto D, Darvishi K, Shi X, Rajan D, Rigler D, Fitzgerald T, et al. Comprehensive assessment of array-based platforms and calling algorithms for detection of copy number variants. Nat Biotechnol 2011 May 8;29(6):512-520.

(2) Pinto D, Pagnamenta AT, Klei L, Anney R, Merico D, Regan R, et al. Functional impact of global rare copy number variation in autism spectrum disorders. Nature 2010 Jul 15;466(7304):368-372.

(3) Winchester L, Yau C, Ragoussis J. Comparing CNV detection methods for SNP arrays. Brief Funct Genomic Proteomic 2009 Sep;8(5):353-366.

(4) Wang K, Li M, Hadley D, Liu R, Glessner J, Grant SF, et al. PennCNV: an integrated hidden Markov model designed for high-resolution copy number variation detection in whole-genome SNP genotyping data. Genome Res 2007 Nov;17(11):1665-1674.

(5) Colella S, Yau C, Taylor JM, Mirza G, Butler H, Clouston P, et al. QuantiSNP: an Objective Bayes Hidden-Markov Model to detect and accurately map copy number variation using SNP genotyping data. Nucleic Acids Res 2007;35(6):2013-2025.

(6) Diskin SJ, Li M, Hou C, Yang S, Glessner J, Hakonarson H, et al. Adjustment of genomic waves in signal intensities from whole-genome SNP genotyping platforms. Nucleic Acids Res 2008 Nov;36(19):e126.

(7) Edgar R, Domrachev M, Lash AE. Gene Expression Omnibus: NCBI gene expression and hybridization array data repository. Nucleic Acids Res 2002 Jan 1;30(1):207-210.

(8) Kent WJ, Sugnet CW, Furey TS, Roskin KM, Pringle TH, Zahler AM, et al. The human genome browser at UCSC. Genome Res 2002 Jun;12(6):996-1006.

(9) Uddin M, Sturge M, Rahman P, Woods MO. Autosome-wide copy number variation association analysis for rheumatoid arthritis using the WTCCC high-density SNP genotype data. J Rheumatol 2011 May;38(5):797-801.

(10) Zheng X, Shaffer JR, McHugh CP, Laurie CC, Feenstra B, Melbye M, et al. Using family data as a verification standard to evaluate copy number variation calling strategies for genetic association studies. Genet Epidemiol 2012 Apr;36(3):253-262.

(11) Lin P, Hartz SM, Wang JC, Krueger RF, Foroud TM, Edenberg HJ, et al. Copy number variation accuracy in genome-wide association studies. Hum Hered 2011;71(3):141-147.

(12) Fernandez-Rozadilla C, Cazier JB, Tomlinson I, Brea-Fernandez A, Lamas MJ, Baiget M, et al. A genome-wide association study on copy-number variation identifies a 11q11 loss as a candidate susceptibility variant for colorectal cancer. Hum Genet 2014 May;133(5):525-534.

(13) Marenne G, Rodriguez-Santiago B, Closas MG, Perez-Jurado L, Rothman N, Rico D, et al. Assessment of copy number variation using the Illumina Infinium 1M SNP-array: a comparison of methodological approaches in the Spanish Bladder Cancer/EPICURO study. Hum Mutat 2011 Feb;32(2):240-248.

(14) Marenne G, Real FX, Rothman N, Rodriguez-Santiago B, Perez-Jurado L, Kogevinas M, et al. Genome-wide CNV analysis replicates the association between GSTM1 deletion and bladder cancer: a support for using continuous measurement from SNP-array data. BMC Genomics 2012 Jul 20;13:326-2164-13-326.

(15) Teo SM, Ku CS, Naidoo N, Hall P, Chia KS, Salim A, et al. A population-based study of copy number variants and regions of homozygosity in healthy Swedish individuals. J Hum Genet 2011 Jul;56(7):524-533.

(16) Tsuang DW, Millard SP, Ely B, Chi P, Wang K, Raskind WH, et al. The effect of algorithms on copy number variant detection. PLoS One 2010 Dec 30;5(12):e14456.

(17) Ukkola-Vuoti L, Kanduri C, Oikkonen J, Buck G, Blancher C, Raijas P, et al. Genome-wide copy number variation analysis in extended families and unrelated individuals characterized for musical aptitude and creativity in music. PLoS One 2013;8(2):e56356.

(18) Jiang L, Jiang J, Yang J, Liu X, Wang J, Wang H, et al. Genome-wide detection of copy number variations using high-density SNP genotyping platforms in Holsteins. BMC Genomics 2013 Feb 27;14:131-2164-14-131.

(19) Nag A, Bochukova EG, Kremeyer B, Campbell DD, Muller H, Valencia-Duarte AV, et al. CNV analysis in Tourette syndrome implicates large genomic rearrangements in COL8A1 and NRXN1. PLoS One 2013;8(3):e59061.

(20) Degenhardt F, Priebe L, Herms S, Mattheisen M, Muhleisen TW, Meier S, et al. Association between copy number variants in 16p11.2 and major depressive disorder in a German case-control sample. Am J Med Genet B Neuropsychiatr Genet 2012 Apr;159B(3):263-273.

(21) Need AC, Ge D, Weale ME, Maia J, Feng S, Heinzen EL, et al. A genome-wide investigation of SNPs and CNVs in schizophrenia. PLoS Genet 2009 Feb;5(2):e1000373.

(22) Priebe L, Degenhardt F, Strohmaier J, Breuer R, Herms S, Witt SH, et al. Copy number variants in German patients with schizophrenia. PLoS One 2013 Jul 2;8(7):e64035.

(23) Purcell S, Neale B, Todd-Brown K, Thomas L, Ferreira MA, Bender D, et al. PLINK: a tool set for whole-genome association and population-based linkage analyses. Am J Hum Genet 2007 Sep;81(3):559-575.

(24) Safran M, Chalifa-Caspi V, Shmueli O, Olender T, Lapidot M, Rosen N, et al. Human Gene-Centric Databases at the Weizmann Institute of Science: GeneCards, UDB, CroW 21 and HORDE. Nucleic Acids Res 2003 Jan 1;31(1):142-146.

(25) Haraksingh RR, Abyzov A, Gerstein M, Urban AE, Snyder M. Genome-wide mapping of copy number variation in humans: comparative analysis of high resolution array platforms. PLoS One 2011;6(11):e27859.

(26) 1000 Genomes Project Consortium, Abecasis GR, Altshuler D, Auton A, Brooks LD, Durbin RM, et al. A map of human genome variation from population-scale sequencing. Nature 2010 Oct 28;467(7319):1061-1073.

(27) Campbell CD, Sampas N, Tsalenko A, Sudmant PH, Kidd JM, Malig M, et al. Population-genetic properties of differentiated human copy-number polymorphisms. Am J Hum Genet 2011 Mar 11;88(3):317-332.

(28) Conrad DF, Pinto D, Redon R, Feuk L, Gokcumen O, Zhang Y, et al. Origins and functional impact of copy number variation in the human genome. Nature 2010 Apr 1;464(7289):704-712.

(29) MacDonald JR, Ziman R, Yuen RK, Feuk L, Scherer SW. The Database of Genomic Variants: a curated collection of structural variation in the human genome. Nucleic Acids Res 2014 Jan;42(Database issue):D986-92.
