# Supplementary material for: Germline INDELs and CNVs in a cohort of colorectal cancer patients: their characteristics, associations with relapse‐free survival time, and potential time‐varying effects on the risk of relapse
Source: Cancer Med. 2017 May 23;6(6):1220–32. doi: 10.1002/cam4.1074 (PMC5463068; doi:10.1002/cam4.1074)
Supplement: Supplementary file 2 — Data S2. Oligonucleotides and amplification conditions for duplex end‐point PCR. [file CAM4-6-1220-s002.docx]

**Supporting Information 2**

**Oligonucleotides and amplification conditions for duplex end-point PCR**

The DNA sequence for each INDEL/CNV was obtained from the UCSC genome browser (1). The repetitive sequences were masked in each DNA sequence using RepeatMasker (<http://www.repeatmasker.org>) (2). These sequences were then used to design primer pairs using the Primer 3 tool under the default conditions (<http://bioinfo.ut.ee/primer3-0.4.0/>) (3). The primer sequences for amplification of a part of the Albumin gene (which was used as the control amplicon in the duplex reactions) were obtained from a previously published study (4). **Supporting Information 2–Table 1** shows the sequences of oligonucleotides, and the size and the genomic coordinates of the amplicons.

Duplex PCRs were performed as follows: 1 µL of genomic DNA (6 ng / µL) was amplified in a 10 µL reaction containing 5 µL of AmpliTaq Gold 360 Master Mix (2X; Applied Biosystems, Foster City, USA), 0.25 µL of 360 GC Enhancer (Applied Biosystems, Foster City, USA), 0.5 µM of each primer (Integrated DNA Technologies, Inc., Coralville, Iowa), and 1.75 µL of sterile water. In cases of poor amplification, PCR was repeated using 12 ng of DNA template. All reactions were carried out in MicroAmp Fast Optical 96-Well Reaction Plates (with barcode, 0.1 mL-Catalogue # 4346906, Applied Biosystems, Foster City, USA) using a Veriti 96-Well Fast Thermal Cycler (Applied Biosystems, Foster City, USA) under the following cycling conditions: 10 minutes at 95°C; 30 cycles of 30 seconds at 95°C, 30 seconds at 57°C, and 60 seconds at 72°C; and finally 7 minutes at 72°C. Non-template controls were included to check for DNA contamination in each reaction mix. In addition to the patient DNA samples, the duplex PCR reactions also included amplification of two commercial DNA samples (Catalogue # G1521 and G1471, Promega, Madison, USA) as controls.

PCR products were analyzed by electrophoresis on 2% - 3% agarose gels stained with SYBR Safe DNA Gel Stain (Invitrogen, Foster City, USA), and visualized using AlphaImager EP (ProteinSimple, San Jose, California).

During this analysis five DNA samples were genotyped twice for each variant; in all cases the genotypes obtained were 100% concordant.

**Supporting Information 2–Table 1.** Oligonucleotides used for amplification of selected INDELs/CNVs.

| **Primer Name** | **Sequence** | **Genomic Location of the Amplicon** | **Amplicon Size (bp)** | **Concordance Rate** | |
| --- | --- | --- | --- | --- | --- |
| *ADAM3A/ADAM5A-F* | 5'-ATC TCT GGG AAA GCC TGG AT-3' | chr8: 39,310,533 - chr8: 39,310,738 | 205 | 98% |  |
| *ADAM3A/ADAM5A-R* | 5'-ACT TAG CTG CCA TTC CCT CA-3' |  |  |  |  |
| *CNOT1-F* | 5'-CCATCAAAAGGGCACTGATT-3' | chr16: 58,647,478 - chr16: 58,647,719 | 241 | 100% |  |
| *CNOT1-R* | 5'-GCGACCAATTTTCTACTTTGA-3' |  |  |  |  |
| *DLEU1-F* | 5'-AGGCTTACTTCCAGGTGCAT-3' | chr13: 51,070,494 - chr13: 51,070,665 | 171 | 100% |  |
| *DLEU1-R* | 5'-TCACCAAGTGGCTACGATCA-3' |  |  |  |  |
| *FAM149A-F* | 5'-CAG TGG CAA AAT CTC CCA AG-3' | chr4: 187,093,696 - chr4: 187,093,942 | 246 | 100% |  |
| *FAM149A-R* | 5'-AAG GTG TCA TTG CAG TGG TG-3' |  |  |  |  |
| *FILIP1L/CMSS1* | 5'-TGGTTTGGGACACACTGACT-3' | chr3: 99,629,157 - chr3: 99,629,378 | 221 | 100% |  |
| *FILIP1L/CMSS1* | 5'-CAACATGCATCTGCCACTTC-3' |  |  |  |  |
| *LCE3C-F* | 5'-AGT TGT CCC TCA CCC AAG TG-3' | chr1: 152,573,251 - chr1: 152,573,402 | 151 | 93% |  |
| *LCE3C-R* | 5'-ATT GAT GGG ACC TGA AGT GC-3' |  |  |  |  |
| *NME7-F* | 5'-AAA TCC AGC ACA GGG ATC TG-3' | chr1: 169,230,753 - chr1: 169,230,911 | 158 | 99% |  |
| *NME7-R* | 5'-TGC CAT CAT CAG AGT CAA GC-3' |  |  |  |  |
| *REV1-F* | 5'-TCG TCT CCT GAC TTG CCT TT-3' | chr2: 100,104,021 - chr2: 100,104,211 | 190 | 100% |  |
| *REV1-R* | 5'-GCA TTG TGG GTC TTT CTG CT-3' |  |  |  |  |
| *WDR34/VTI1BP4-F* | 5'-TGGCTTTCACTTGGCTTTCT-3' | chr9: 131,412,585 - chr9: 131,412,785 | 200 | 100% |  |
| *WDR34/VTI1BP4-F* | 5'-TTTCTTGCCACGTCCCTATC-3' |  |  |  |  |
| *WWOX-F* | 5'-ATG TCA GTG TCC CCC ACA AT-3' | chr16: 78,381,127 - chr16: 78,381,337 | 210 | 100% |  |
| *WWOX-R* | 5'-GTC AAG AGT GCT GTG CCA AA-3' |  |  |  |  |

bp: base pair; chr: chromosome; F: forward; R: reverse. Genomic coordinates are based on hg19.

**References**

(1) Kent WJ, Sugnet CW, Furey TS, Roskin KM, Pringle TH, Zahler AM, et al. The human genome browser at UCSC. Genome Res 2002 Jun;12(6):996-1006.

(2) Smit AFA, Hubley R, Green P. *RepeatMasker Open-4.0*. 2013-2015.

(3) Untergasser A, Cutcutache I, Koressaar T, Ye J, Faircloth BC, Remm M, et al. Primer3--new capabilities and interfaces. Nucleic Acids Res 2012 Aug;40(15):e115.

(4) Arand M, Mühlbauer R, Hengstler J, Jäger E, Fuchs J, Winkler L, et al. A Multiplex Polymerase Chain Reaction Protocol for the Simultaneous Analysis of the GlutathioneS-Transferase GSTM1 and GSTT1 Polymorphisms. Anal Biochem 1996 4/5;236(1):184-186.
