# Supplementary material for: Germline INDELs and CNVs in a cohort of colorectal cancer patients: their characteristics, associations with relapse‐free survival time, and potential time‐varying effects on the risk of relapse
Source: Cancer Med. 2017 May 23;6(6):1220–32. doi: 10.1002/cam4.1074 (PMC5463068; doi:10.1002/cam4.1074)
Supplement: Supplementary file 3 — Data S3. Information on the 106 INDELs/CNVs. [file CAM4-6-1220-s003.docx]

**Supporting Information 3**

**Information on the 106 INDELs/CNVs.**

**Supporting Information 3 – Table 1.** Information on the 106 INDELs/CNVs.

| **Variant type** | **Length** | **CHR** | **Start** | **End** | **CN state** | **CN freq.** | **Gene** | **Ensembl ID** | **% Overlap** | **Overlap type** |
| --- | --- | --- | --- | --- | --- | --- | --- | --- | --- | --- |
| INDEL | 906 | 1 | 17676291 | 17677196 | 0 | 0.109 | *PADI4* | ENSG00000159339 | 100.0 | Variant located inside the gene |
| INDEL | 521 | 1 | 58744143 | 58744663 | 0 | 0.762 | *DAB1* | ENSG00000173406 | 100.0 | Variant located inside the gene |
| CNV | 45280 | 1 | 72766413 | 72811692 | 0 | 0.343 | *RPL31P12* | ENSG00000227207 | 0.8 | Variant covers the whole gene |
| CNV | 2006 | 1 | 89476427 | 89478432 | 0 | 0.103 | *GBP3* | ENSG00000117226 | 100.0 | Variant located inside the gene |
| CNV | 1117 | 1 | 92232111 | 92233227 | 0 | 0.101 | *TGFBR3* | ENSG00000069702 | 100.0 | Variant located inside the gene |
| CNV | 30855 | 1 | 152556085 | 152586939 | 0 | 0.331 | *LCE3C* | ENSG00000244057 | 1.4 | Variant covers the whole gene |
| CNV | 30855 | 1 | 152556085 | 152586939 | 0 | 0.331 | *LCE3B* | ENSG00000187238 | 0.9 | Variant covers the whole gene |
| CNV | 30855 | 1 | 152556085 | 152586939 | 3 | 0.016 | *LCE3C* | ENSG00000244057 | 1.4 | Variant covers the whole gene |
| CNV | 30855 | 1 | 152556085 | 152586939 | 3 | 0.016 | *LCE3B* | ENSG00000187238 | 0.9 | Variant covers the whole gene |
| CNV | 33950 | 1 | 169207360 | 169241309 | 0 | 0.117 | *NME7* | ENSG00000143156 | 100.0 | Variant located inside the gene |
| CNV | 33950 | 1 | 169207360 | 169241309 | 1 | 0.333 | *NME7* | ENSG00000143156 | 100.0 | Variant located inside the gene |
| INDEL | 555 | 1 | 179607382 | 179607936 | 0 | 0.129 | *TDRD5* | ENSG00000162782 | 100.0 | Variant located inside the gene |
| INDEL | 601 | 1 | 207292578 | 207293178 | 0 | 0.453 | *C4BPA* | ENSG00000123838 | 100.0 | Variant located inside the gene |
| CNV | 2583 | 2 | 33224605 | 33227187 | 0 | 0.101 | *LTBP1* | ENSG00000049323 | 100.0 | Variant located inside the gene |
| CNV | 38030 | 2 | 34698447 | 34736476 | 0 | 0.147 | *AC073218.1* | ENSG00000226785 | 100.0 | Variant located inside the gene |
| CNV | 38030 | 2 | 34698447 | 34736476 | 1 | 0.220 | *AC073218.1* | ENSG00000226785 | 100.0 | Variant located inside the gene |
| CNV | 1713 | 2 | 54565729 | 54567441 | 0 | 0.323 | *C2orf73* | ENSG00000177994 | 100.0 | Variant located inside the gene |
| CNV | 1862 | 2 | 54565729 | 54567590 | 0 | 0.280 | *C2orf73* | ENSG00000177994 | 100.0 | Variant located inside the gene |
| CNV | 1862 | 2 | 54565729 | 54567590 | 1 | 0.020 | *C2orf73* | ENSG00000177994 | 100.0 | Variant located inside the gene |
| INDEL | 413 | 2 | 70125092 | 70125504 | 0 | 0.152 | *SNRNP27* | ENSG00000124380 | 100.0 | Variant located inside the gene |
| INDEL | 413 | 2 | 70125092 | 70125504 | 0 | 0.152 | *MXD1* | ENSG00000059728 | 100.0 | Variant located inside the gene |
| CNV | 1262 | 2 | 100103752 | 100105013 | 0 | 0.200 | *REV1* | ENSG00000135945 | 100.0 | Variant located inside the gene |
| CNV | 1428 | 2 | 159959587 | 159961014 | 0 | 0.305 | *TANC1* | ENSG00000115183 | 100.0 | Variant located inside the gene |
| CNV | 1865 | 2 | 159959587 | 159961451 | 0 | 0.176 | *TANC1* | ENSG00000115183 | 100.0 | Variant located inside the gene |
| INDEL | 540 | 2 | 182856938 | 182857477 | 0 | 0.111 | *PPP1R1C* | ENSG00000150722 | 100.0 | Variant located inside the gene |
| CNV | 1844 | 2 | 215728845 | 215730688 | 0 | 0.137 | *AC072062.1* | ENSG00000229267 | 100.0 | Variant located inside the gene |
| CNV | 4671 | 3 | 32102055 | 32106725 | 0 | 0.196 | *NIFKP7* | ENSG00000251590 | 27.0 | Variant partially overlaps with the gene |
| CNV | 4671 | 3 | 32102055 | 32106725 | 0 | 0.196 | *OSBPL10* | ENSG00000144645 | 100.0 | Variant located inside the gene |
| CNV | 4671 | 3 | 32102055 | 32106725 | 1 | 0.002 | *NIFKP7* | ENSG00000251590 | 27.0 | Variant partially overlaps with the gene |
| CNV | 4671 | 3 | 32102055 | 32106725 | 1 | 0.002 | *OSBPL10* | ENSG00000144645 | 100.0 | Variant located inside the gene |
| CNV | 2627 | 3 | 47490712 | 47493338 | 0 | 0.141 | *SCAP* | ENSG00000114650 | 100.0 | Variant located inside the gene |
| INDEL | 746 | 3 | 99628822 | 99629567 | 0 | 0.103 | *CMSS1* | ENSG00000184220 | 100.0 | Variant located inside the gene |
| INDEL | 746 | 3 | 99628822 | 99629567 | 0 | 0.103 | *FILIP1L* | ENSG00000168386 | 100.0 | Variant located inside the gene |
| INDEL | 746 | 3 | 99628822 | 99629567 | 1 | 0.158 | *CMSS1* | ENSG00000184220 | 100.0 | Variant located inside the gene |
| INDEL | 746 | 3 | 99628822 | 99629567 | 1 | 0.158 | *FILIP1L* | ENSG00000168386 | 100.0 | Variant located inside the gene |
| CNV | 2092 | 3 | 107038162 | 107040253 | 0 | 0.101 | *LINC00883* | ENSG00000243701 | 100.0 | Variant located inside the gene |
| CNV | 2092 | 3 | 107038162 | 107040253 | 0 | 0.101 | *RP11-446H18.5* | ENSG00000239828 | 100.0 | Variant T located inside the gene |
| INDEL | 541 | 3 | 124936371 | 124936911 | 0 | 0.166 | *SLC12A8* | ENSG00000221955 | 100.0 | Variant located inside the gene |
| INDEL | 541 | 3 | 124936371 | 124936911 | 1 | 0.004 | *SLC12A8* | ENSG00000221955 | 100.0 | Variant located inside the gene |
| CNV | 4666 | 3 | 131708352 | 131713017 | 0 | 0.123 | *CPNE4* | ENSG00000196353 | 100.0 | Variant located inside the gene |
| CNV | 4666 | 3 | 131708352 | 131713017 | 1 | 0.044 | *CPNE4* | ENSG00000196353 | 100.0 | Variant located inside the gene |
| CNV | 5050 | 3 | 136021052 | 136026101 | 0 | 0.129 | *PCCB* | ENSG00000114054 | 100.0 | Variant located inside the gene |
| CNV | 5050 | 3 | 136021052 | 136026101 | 1 | 0.004 | *PCCB* | ENSG00000114054 | 100.0 | Variant located inside the gene |
| INDEL | 554 | 3 | 159257057 | 159257610 | 0 | 0.123 | *IQCJ-SCHIP1* | ENSG00000250588 | 100.0 | Variant located inside the gene |
| CNV | 3201 | 3 | 162765807 | 162769007 | 0 | 0.521 | *RP11-10O22.1* | ENSG00000241168 | 100.0 | Variant located inside the gene |
| CNV | 3087 | 3 | 189737354 | 189740440 | 0 | 0.101 | *LEPREL1* | ENSG00000090530 | 100.0 | Variant located inside the gene |
| CNV | 3087 | 3 | 189737354 | 189740440 | 1 | 0.006 | *LEPREL1* | ENSG00000090530 | 100.0 | Variant located inside the gene |
| CNV | 9416 | 3 | 192875738 | 192885153 | 0 | 0.483 | *RP11-143P4.2* | ENSG00000232130 | 100.0 | Variant located inside the gene |
| CNV | 9416 | 3 | 192875738 | 192885153 | 4 | 0.002 | *RP11-143P4.2* | ENSG00000232130 | 100.0 | Variant located inside the gene |
| CNV | 2737 | 4 | 91933043 | 91935779 | 0 | 0.141 | *CCSER1* | ENSG00000184305 | 100.0 | Variant located inside the gene |
| INDEL | 533 | 4 | 115928747 | 115929279 | 0 | 0.105 | *NDST4* | ENSG00000138653 | 100.0 | Variant located inside the gene |
| INDEL | 647 | 4 | 138966505 | 138967151 | 0 | 0.133 | *LINC00616* | ENSG00000248307 | 100.0 | Variant located inside the gene |
| CNV | 1121 | 4 | 146438871 | 146439991 | 0 | 0.164 | *SMAD1* | ENSG00000170365 | 100.0 | Variant located inside the gene |
| CNV | 1121 | 4 | 146438871 | 146439991 | 1 | 0.022 | *SMAD1* | ENSG00000170365 | 100.0 | Variant located inside the gene |
| CNV | 1187 | 4 | 166003471 | 166004657 | 0 | 0.776 | *TMEM192* | ENSG00000170088 | 100.0 | Variant located inside the gene |
| CNV | 3802 | 4 | 172989075 | 172992876 | 0 | 0.216 | *GALNTL6* | ENSG00000174473 | 100.0 | Variant located inside the gene |
| INDEL | 501 | 4 | 182056607 | 182057107 | 0 | 0.309 | *LINC00290* | ENSG00000248197 | 100.0 | Variant located inside the gene |
| CNV | 2092 | 4 | 186441932 | 186444023 | 0 | 0.564 | *PDLIM3* | ENSG00000154553 | 100.0 | Variant located inside the gene |
| CNV | 2179 | 4 | 186441932 | 186444110 | 0 | 0.204 | *PDLIM3* | ENSG00000154553 | 100.0 | Variant located inside the gene |
| CNV | 4515 | 4 | 187093557 | 187098071 | 0 | 0.313 | *FAM149A* | ENSG00000109794 | 5.8 | Variant partially overlaps with the gene |
| CNV | 1915 | 5 | 1178511 | 1180425 | 0 | 0.180 | *CTD-3080P12.3* | ENSG00000249201 | 10.9 | partially overlaps with the gene |
| CNV | 1648 | 5 | 21450792 | 21452439 | 0 | 0.139 | *GUSBP1* | ENSG00000183666 | 100.0 | Variant located inside the gene |
| CNV | 1498 | 5 | 106324802 | 106326299 | 0 | 0.180 | *CTC-254B4.1* | ENSG00000251027 | 100.0 | Variant located inside the gene |
| CNV | 1001 | 5 | 147553186 | 147554186 | 0 | 0.327 | *SPINK14* | ENSG00000196800 | 100.0 | Variant located inside the gene |
| CNV | 1222 | 6 | 666535 | 667756 | 0 | 0.388 | *EXOC2* | ENSG00000112685 | 100.0 | Variant located inside the gene |
| INDEL | 501 | 6 | 18402172 | 18402672 | 0 | 0.402 | *snoU13* | ENSG00000238458 | 20.2 | Variant covers the whole gene |
| INDEL | 501 | 6 | 18402172 | 18402672 | 0 | 0.402 | *RNF144B* | ENSG00000137393 | 100.0 | Variant located inside the gene |
| INDEL | 568 | 6 | 51736175 | 51736742 | 0 | 0.251 | *PKHD1* | ENSG00000170927 | 100.0 | Variant located inside the gene |
| INDEL | 568 | 6 | 51736175 | 51736742 | 1 | 0.057 | *PKHD1* | ENSG00000170927 | 100.0 | Variant located inside the gene |
| CNV | 4098 | 6 | 53929777 | 53933874 | 0 | 0.473 | *MLIP-AS1* | ENSG00000235050 | 100.0 | Variant located inside the gene |
| CNV | 4098 | 6 | 53929777 | 53933874 | 0 | 0.473 | *MLIP* | ENSG00000146147 | 100.0 | Variant located inside the gene |
| CNV | 1627 | 6 | 65347533 | 65349159 | 0 | 0.289 | *EYS* | ENSG00000188107 | 100.0 | Variant located inside the gene |
| INDEL | 390 | 6 | 89921782 | 89922171 | 0 | 0.317 | *GABRR1* | ENSG00000146276 | 100.0 | Variant located inside the gene |
| INDEL | 880 | 6 | 167488211 | 167489090 | 0 | 0.176 | *RP11-517H2.6* | ENSG00000272980 | 100.0 | Variant located inside the gene |
| CNV | 1389 | 7 | 89810608 | 89811996 | 0 | 0.131 | *STEAP2-AS1* | ENSG00000227646 | 100.0 | Variant located inside the gene |
| CNV | 1389 | 7 | 89810608 | 89811996 | 0 | 0.131 | *STEAP2* | ENSG00000157214 | 100.0 | Variant located inside the gene |
| CNV | 1507 | 7 | 89810608 | 89812114 | 0 | 0.135 | *STEAP2-AS1* | ENSG00000227646 | 100.0 | Variant located inside the gene |
| CNV | 1507 | 7 | 89810608 | 89812114 | 0 | 0.135 | *STEAP2* | ENSG00000157214 | 100.0 | Variant located inside the gene |
| CNV | 2798 | 7 | 126048572 | 126051369 | 0 | 0.121 | *AC000370.2* | ENSG00000241921 | 100.0 | Variant located inside the gene |
| CNV | 2905 | 7 | 126048572 | 126051476 | 0 | 0.178 | *AC000370.2* | ENSG00000241921 | 100.0 | Variant located inside the gene |
| CNV | 1888 | 7 | 148074379 | 148076266 | 0 | 0.675 | *CNTNAP2* | ENSG00000174469 | 100.0 | Variant located inside the gene |
| CNV | 4441 | 8 | 594761 | 599201 | 0 | 0.677 | *ERICH1* | ENSG00000104714 | 100.0 | Variant located inside the gene |
| CNV | 1196 | 8 | 4122961 | 4124156 | 0 | 0.426 | *CSMD1* | ENSG00000183117 | 100.0 | Variant located inside the gene |
| CNV | 1409 | 8 | 11245641 | 11247049 | 0 | 0.176 | *C8orf12* | ENSG00000184608 | 100.0 | Variant located inside the gene |
| CNV | 3753 | 8 | 25066884 | 25070636 | 0 | 0.596 | *DOCK5* | ENSG00000147459 | 100.0 | Variant located inside the gene |
| CNV | 153836 | 8 | 39233344 | 39387179 | 0 | 0.172 | *ADAM5* | ENSG00000196115 | 27.0 | Variant partially overlaps with the gene |
| CNV | 153836 | 8 | 39233344 | 39387179 | 0 | 0.172 | *ADAM3A* | ENSG00000197475 | 46.7 | Variant covers the whole gene |
| CNV | 153836 | 8 | 39233344 | 39387179 | 1 | 0.343 | *ADAM5* | ENSG00000196115 | 27.0 | Variant partially overlaps with the gene |
| CNV | 153836 | 8 | 39233344 | 39387179 | 1 | 0.343 | *ADAM3A* | ENSG00000197475 | 46.7 | Variant covers the whole gene |
| CNV | 2303 | 8 | 75364528 | 75366830 | 0 | 0.349 | *GDAP1* | ENSG00000104381 | 100.0 | Variant located inside the gene |
| CNV | 3498 | 8 | 137160319 | 137163816 | 0 | 0.265 | *RP11-149P24.1* | ENSG00000253248 | 100.0 | Variant located inside the gene |
| CNV | 1884 | 9 | 71741217 | 71743100 | 0 | 0.527 | *TJP2* | ENSG00000119139 | 100.0 | Variant located inside the gene |
| CNV | 2022 | 9 | 101309058 | 101311079 | 0 | 0.240 | *GABBR2* | ENSG00000136928 | 100.0 | Variant located inside the gene |
| CNV | 1305 | 9 | 131412549 | 131413853 | 0 | 0.323 | *VTI1BP4* | ENSG00000227759 | 44.2 | Variant partially overlaps with the gene |
| CNV | 1305 | 9 | 131412549 | 131413853 | 0 | 0.323 | *WDR34* | ENSG00000119333 | 100.0 | Variant located inside the gene |
| CNV | 1337 | 9 | 131412549 | 131413885 | 0 | 0.493 | *VTI1BP4* | ENSG00000227759 | 45.5 | Variant partially overlaps with the gene |
| CNV | 1337 | 9 | 131412549 | 131413885 | 0 | 0.493 | *WDR34* | ENSG00000119333 | 100.0 | Variant located inside the gene |
| INDEL | 969 | 9 | 138479177 | 138480145 | 0 | 0.362 | *RP11-98L5.4* | ENSG00000224045 | 30.7 | Variant partially overlaps with the gene |
| CNV | 1672 | 10 | 4708627 | 4710298 | 0 | 0.533 | *LINC00704* | ENSG00000231298 | 100.0 | Variant located inside the gene |
| CNV | 1257 | 10 | 27000558 | 27001814 | 0 | 0.352 | *PDSS1* | ENSG00000148459 | 100.0 | Variant located inside the gene |
| CNV | 4822 | 10 | 78255873 | 78260694 | 0 | 0.648 | *C10orf11* | ENSG00000148655 | 100.0 | Variant located inside the gene |
| INDEL | 520 | 10 | 89275888 | 89276407 | 0 | 0.430 | *MINPP1* | ENSG00000107789 | 100.0 | Variant located inside the gene |
| INDEL | 520 | 10 | 89275888 | 89276407 | 1 | 0.002 | *MINPP1* | ENSG00000107789 | 100.0 | Variant located inside the gene |
| INDEL | 738 | 10 | 95545536 | 95546273 | 0 | 0.891 | *LGI1* | ENSG00000108231 | 100.0 | Variant located inside the gene |
| CNV | 2987 | 10 | 114113589 | 114116575 | 0 | 0.129 | *GUCY2GP* | ENSG00000243316 | 91.9 | Variant partially overlaps with the gene |
| CNV | 1588 | 10 | 122226947 | 122228534 | 0 | 0.145 | *PPAPDC1A* | ENSG00000203805 | 100.0 | Variant located inside the gene |
| CNV | 2181 | 11 | 5760106 | 5762286 | 0 | 0.135 | *TRIM5* | ENSG00000132256 | 100.0 | Variant located inside the gene |
| INDEL | 472 | 11 | 9324025 | 9324496 | 0 | 0.230 | *TMEM41B* | ENSG00000166471 | 100.0 | Variant located inside the gene |
| CNV | 3369 | 11 | 31394060 | 31397428 | 0 | 0.133 | *DNAJC24* | ENSG00000170946 | 100.0 | Variant located inside the gene |
| CNV | 3369 | 11 | 31394060 | 31397428 | 1 | 0.117 | *DNAJC24* | ENSG00000170946 | 100.0 | Variant located inside the gene |
| CNV | 1005 | 11 | 45430401 | 45431405 | 0 | 0.297 | *RP11-430H10.4* | ENSG00000255041 | 100.0 | Variant located inside the gene |
| INDEL | 877 | 11 | 66712229 | 66713105 | 0 | 0.232 | *PC* | ENSG00000173599 | 100.0 | Variant located inside the gene |
| INDEL | 432 | 12 | 12026506 | 12026937 | 0 | 0.121 | *ETV6* | ENSG00000139083 | 100.0 | Variant located inside the gene |
| INDEL | 760 | 12 | 16420184 | 16420943 | 0 | 0.182 | *SLC15A5* | ENSG00000188991 | 100.0 | Variant located inside the gene |
| CNV | 6414 | 12 | 45903118 | 45909531 | 0 | 0.289 | *RP11-352M15.1* | ENSG00000257657 | 100.0 | Variant located inside the gene |
| CNV | 6414 | 12 | 45903118 | 45909531 | 1 | 0.065 | *RP11-352M15.1* | ENSG00000257657 | 100.0 | Variant located inside the gene |
| INDEL | 601 | 13 | 39934551 | 39935151 | 0 | 0.366 | *LHFP* | ENSG00000183722 | 100.0 | Variant located inside the gene |
| CNV | 3249 | 13 | 51069352 | 51072600 | 0 | 0.535 | *DLEU1* | ENSG00000176124 | 100.0 | Variant located inside the gene |
| CNV | 2194 | 13 | 101894125 | 101896318 | 0 | 0.305 | *NALCN* | ENSG00000102452 | 100.0 | Variant located inside the gene |
| INDEL | 623 | 15 | 39372623 | 39373245 | 0 | 0.172 | *RP11-624L4.1* | ENSG00000259345 | 100.0 | Variant located inside the gene |
| INDEL | 953 | 15 | 71881673 | 71882625 | 0 | 0.101 | *THSD4* | ENSG00000187720 | 100.0 | Variant located inside the gene |
| CNV | 3844 | 15 | 76891342 | 76895185 | 0 | 0.253 | *SCAPER* | ENSG00000140386 | 100.0 | Variant located inside the gene |
| CNV | 3844 | 15 | 76891342 | 76895185 | 1 | 0.012 | *SCAPER* | ENSG00000140386 | 100.0 | Variant located inside the gene |
| CNV | 1497 | 15 | 91981864 | 91983360 | 0 | 0.283 | *RP11-661P17.1* | ENSG00000258551 | 100.0 | Variant located inside the gene |
| CNV | 2252 | 16 | 58647399 | 58649650 | 0 | 0.234 | *CNOT1* | ENSG00000125107 | 100.0 | Variant located inside the gene |
| CNV | 3386 | 16 | 76540062 | 76543447 | 0 | 0.499 | *CNTNAP4* | ENSG00000152910 | 100.0 | Variant located inside the gene |
| CNV | 11036 | 16 | 78373700 | 78384735 | 0 | 0.242 | *WWOX* | ENSG00000186153 | 100.0 | Variant located inside the gene |
| CNV | 11036 | 16 | 78373700 | 78384735 | 1 | 0.067 | *WWOX* | ENSG00000186153 | 100.0 | Variant located inside the gene |
| INDEL | 360 | 17 | 724239 | 724598 | 0 | 0.345 | *NXN* | ENSG00000167693 | 100.0 | Variant located inside the gene |
| CNV | 2782 | 17 | 35755867 | 35758648 | 0 | 0.145 | *ACACA* | ENSG00000132142 | 100.0 | Variant located inside the gene |
| CNV | 2782 | 17 | 35755867 | 35758648 | 1 | 0.010 | *ACACA* | ENSG00000132142 | 100.0 | Variant located inside the gene |
| CNV | 1677 | 17 | 55688120 | 55689796 | 0 | 0.360 | *MSI2* | ENSG00000153944 | 100.0 | Variant located inside the gene |
| INDEL | 518 | 18 | 24571673 | 24572190 | 0 | 0.168 | *AQP4-AS1* | ENSG00000260372 | 100.0 | Variant located inside the gene |
| INDEL | 518 | 18 | 24571673 | 24572190 | 0 | 0.168 | *CHST9* | ENSG00000154080 | 100.0 | Variant located inside the gene |
| CNV | 3166 | 18 | 47695103 | 47698268 | 0 | 0.107 | *MYO5B* | ENSG00000167306 | 100.0 | Variant located inside the gene |
| INDEL | 930 | 18 | 75267039 | 75267968 | 0 | 0.325 | *RP11-176N18.2* | ENSG00000264015 | 100.0 | Variant located inside the gene |
| INDEL | 727 | 19 | 2909643 | 2910369 | 0 | 0.794 | *ZNF57* | ENSG00000171970 | 100.0 | Variant located inside the gene |
| CNV | 2427 | 19 | 12694963 | 12697389 | 0 | 0.483 | *ZNF490* | ENSG00000188033 | 100.0 | Variant located inside the gene |
| CNV | 1676 | 21 | 19327135 | 19328810 | 0 | 0.105 | *CHODL* | ENSG00000154645 | 100.0 | Variant located inside the gene |
| CNV | 1676 | 21 | 19327135 | 19328810 | 1 | 0.184 | *CHODL* | ENSG00000154645 | 100.0 | Variant located inside the gene |
| CNV | 2812 | 21 | 44970373 | 44973184 | 0 | 0.303 | *RPL31P1* | ENSG00000214326 | 13.2 | Variant covers the whole gene |
| CNV | 2812 | 21 | 44970373 | 44973184 | 0 | 0.303 | *HSF2BP* | ENSG00000160207 | 100.0 | Variant located inside the gene |
| CNV | 1664 | 22 | 18058001 | 18059664 | 0 | 0.796 | *SLC25A18* | ENSG00000182902 | 100.0 | Variant located inside the gene |
| CNV | 2471 | 22 | 24365041 | 24367511 | 0 | 0.139 | *AP000351.9* | ENSG00000184490 | 30.8 | Variant partially overlaps with the gene |
| INDEL | 529 | 22 | 35645524 | 35646052 | 0 | 0.428 | *RNU7-167P* | ENSG00000238584 | 11.6 | Variant covers the whole gene |

CHR: chromosome; CN: copy number state; freq: frequency
