# Supplementary material for: Germline INDELs and CNVs in a cohort of colorectal cancer patients: their characteristics, associations with relapse‐free survival time, and potential time‐varying effects on the risk of relapse
Source: Cancer Med. 2017 May 23;6(6):1220–32. doi: 10.1002/cam4.1074 (PMC5463068; doi:10.1002/cam4.1074)
Supplement: Supplementary file 4 — Data S4. Summary of statistical analyses. [file CAM4-6-1220-s004.docx]

**Supporting Information 4**

**Summary of statistical analyses.**

**Supporting Information 4 - Table 1.** Multivariable Cox PH (proportional hazards) regression analysis results assuming that all variables satisfy the PH assumption

| **Gene** | **INDEL/CNV** | **p-value** | **HR** | **95% CI (lower)** | **95% CI (higher)** |
| --- | --- | --- | --- | --- | --- |
| *PADI4* | CHR_1_17676291_17677196 (0 copy vs 2 copy) | 0.786 | 1.067 | 0.669 | 1.701 |
| *DAB1* | CHR_1_58744143_58744663 (0 copy vs 2 copy) | 0.92 | 0.983 | 0.7 | 1.379 |
| *RPL31P12* | CHR_1_72766413_72811692 (0 copy vs 2 copy) | 0.846 | 0.97 | 0.716 | 1.315 |
| *GBP3* | CHR_1_89476427_89478432 (0 copy vs 2 copy) | 0.89 | 1.033 | 0.652 | 1.638 |
| *TGFBR3* | CHR_1_92232111_92233227 (0 copy vs 2 copy) | **0.033** | 0.5 | 0.264 | 0.946 |
| *LCE3C, LCE3B* | CHR_1_152556085_152586939 (0 copy vs 2 or 3 copy) | 0.942 | 0.988 | 0.719 | 1.359 |
| *NME7* | CHR_1_169207360_169241309 (0 copy vs 1 or 2 copy) | 0.975 | 0.992 | 0.615 | 1.602 |
| *TDRD5* | CHR_1_179607382_179607936 (0 copy vs 2 copy) | 0.701 | 1.087 | 0.712 | 1.659 |
| *C4BPA* | CHR_1_207292578_207293178 (0 copy vs 2 copy) | 0.647 | 1.069 | 0.802 | 1.425 |
| *LTBP1* | CHR_2_33224605_33227187 (0 copy vs 2 copy) | 0.737 | 1.083 | 0.681 | 1.723 |
| *AC073218.1* | CHR_2_34698447_34736476 (0 copy vs 1 or 2 copy) | 0.172 | 1.306 | 0.89 | 1.916 |
| *C2orf73* | CHR_2_54565729_54567441 (0 copy vs 2 copy) | 0.637 | 0.929 | 0.685 | 1.26 |
| *C2orf73* | CHR_2_54565729_54567590 (0 copy vs 1 or 2 copy) | 0.57 | 0.908 | 0.651 | 1.267 |
| *SNRNP27, MXD1* | CHR_2_70125092_70125504 (0 copy vs 2 copy) | 0.313 | 0.805 | 0.529 | 1.226 |
| *REV1* | CHR_2_100103752_100105013 (0 copy vs 2 copy) | 0.158 | 1.284 | 0.908 | 1.817 |
| *TANC1* | CHR_2_159959587_159961014 (0 copy vs 2 copy) | 0.264 | 0.832 | 0.602 | 1.149 |
| *TANC1* | CHR_2_159959587_159961451 (0 copy vs 2 copy) | 0.747 | 0.936 | 0.628 | 1.396 |
| *PPP1R1C* | CHR_2_182856938_182857477 (0 copy vs 2 copy) | 0.446 | 1.187 | 0.764 | 1.843 |
| *AC072062.1* | CHR_2_215728845_215730688 (0 copy vs 2 copy) | 0.965 | 0.991 | 0.644 | 1.523 |
| *NIFKP7, OSBPL10* | CHR_3_32102055_32106725 (0 copy vs 1 or 2 copy) | 0.469 | 1.138 | 0.802 | 1.616 |
| *SCAP* | CHR_3_47490712_47493338 (0 copy vs 2 copy) | 0.211 | 0.769 | 0.509 | 1.161 |
| *CMSS1, FILIP1L* | CHR_3_99628822_99629567 (0 copy vs 1 or 2 copy) | **0.019** | 1.661 | 1.087 | 2.536 |
| *LINC00883, RP11-446H18.5* | CHR_3_107038162_107040253 (0 copy vs 2 copy) | 0.848 | 0.956 | 0.605 | 1.512 |
| *SLC12A8* | CHR_3_124936371_124936911 (0 copy vs 1 or 2 copy) | 0.853 | 1.036 | 0.713 | 1.504 |
| *CPNE4* | CHR_3_131708352_131713017 (0 copy vs 1 or 2 copy) | 0.173 | 1.325 | 0.884 | 1.985 |
| *PCCB* | CHR_3_136021052_136026101 (0 copy vs 1 or 2 copy) | 0.659 | 1.102 | 0.716 | 1.696 |
| *IQCJ-SCHIP1* | CHR_3_159257057_159257610 (0 copy vs 2 copy) | 0.514 | 1.161 | 0.742 | 1.815 |
| *RP11-10O22.1* | CHR_3_162765807_162769007 (0 copy vs 2 copy) | 0.792 | 0.962 | 0.722 | 1.282 |
| *LEPREL1* | CHR_3_189737354_189740440 (0 copy vs 1 or 2 copy) | 0.096 | 1.441 | 0.938 | 2.215 |
| *RP11-143P4.2* | CHR_3_192875738_192885153 (0 copy vs 2 or 4 copy) | 0.059 | 1.321 | 0.99 | 1.764 |
| *CCSER1* | CHR_4_91933043_91935779 (0 copy vs 2 copy) | 0.719 | 0.922 | 0.593 | 1.435 |
| *NDST4* | CHR_4_115928747_115929279 (0 copy vs 2 copy) | 0.111 | 0.632 | 0.359 | 1.112 |
| *LINC00616* | CHR_4_138966505_138967151 (0 copy vs 2 copy) | 0.82 | 0.95 | 0.612 | 1.475 |
| *SMAD1* | CHR_4_146438871_146439991 (0 copy vs 1 or 2 copy) | 0.94 | 1.015 | 0.682 | 1.511 |
| *TMEM192* | CHR_4_166003471_166004657 (0 copy vs 2 copy) | 0.349 | 1.183 | 0.832 | 1.683 |
| *GALNTL6* | CHR_4_172989075_172992876 (0 copy vs 2 copy) | 0.842 | 1.035 | 0.737 | 1.453 |
| *LINC00290* | CHR_4_182056607_182057107 (0 copy vs 2 copy) | 0.926 | 1.015 | 0.744 | 1.384 |
| *PDLIM3* | CHR_4_186441932_186444023 (0 copy vs 2 copy) | 0.125 | 0.799 | 0.599 | 1.065 |
| *PDLIM3* | CHR_4_186441932_186444110 (0 copy vs 2 copy) | 0.186 | 1.265 | 0.893 | 1.794 |
| *FAM149A* | CHR_4_187093557_187098071 (0 copy vs 2 copy) | 0.831 | 1.035 | 0.756 | 1.415 |
| *CTD-3080P12.3* | CHR_5_1178511_1180425 (0 copy vs 2 copy) | 0.177 | 1.269 | 0.898 | 1.793 |
| *GUSBP1* | CHR_5_21450792_21452439 (0 copy vs 2 copy) | 0.832 | 0.956 | 0.634 | 1.442 |
| *CTC-254B4.1* | CHR_5_106324802_106326299 (0 copy vs 2 copy) | 0.943 | 1.014 | 0.702 | 1.464 |
| *SPINK14* | CHR_5_147553186_147554186 (0 copy vs 2 copy) | 0.764 | 0.954 | 0.703 | 1.296 |
| *EXOC2* | CHR_6_666535_667756 (0 copy vs 2 copy) | 0.927 | 0.986 | 0.734 | 1.326 |
| *snoU13, RNF144B* | CHR_6_18402172_18402672 (0 copy vs 2 copy) | 0.366 | 1.143 | 0.855 | 1.529 |
| *PKHD1* | CHR_6_51736175_51736742 (0 copy vs 1 or 2 copy) | 0.211 | 1.226 | 0.891 | 1.689 |
| *MLIP-AS1, MLIP* | CHR_6_53929777_53933874 (0 copy vs 2 copy) | 0.235 | 0.839 | 0.628 | 1.121 |
| *EYS* | CHR_6_65347533_65349159 (0 copy vs 2 copy) | 0.466 | 0.89 | 0.65 | 1.219 |
| *GABRR1* | CHR_6_89921782_89922171 (0 copy vs 2 copy) | 0.284 | 1.178 | 0.873 | 1.591 |
| *RP11-517H2.6* | CHR_6_167488211_167489090 (0 copy vs 2 copy) | 0.516 | 1.132 | 0.778 | 1.648 |
| *STEAP2-AS1, STEAP2* | CHR_7_89810608_89811996 (0 copy vs 2 copy) | 0.638 | 0.891 | 0.551 | 1.441 |
| *STEAP2-AS1, STEAP2* | CHR_7_89810608_89812114 (0 copy vs 2 copy) | **0.031** | 0.568 | 0.34 | 0.95 |
| *AC000370.2* | CHR_7_126048572_126051369 (0 copy vs 2 copy) | 0.444 | 0.826 | 0.506 | 1.348 |
| *AC000370.2* | CHR_7_126048572_126051476 (0 copy vs 2 copy) | 0.739 | 0.937 | 0.639 | 1.374 |
| *CNTNAP2* | CHR_7_148074379_148076266 (0 copy vs 2 copy) | 0.395 | 0.876 | 0.647 | 1.188 |
| *ERICH1* | CHR_8_594761_599201 (0 copy vs 2 copy) | 0.874 | 0.976 | 0.72 | 1.322 |
| *CSMD1* | CHR_8_4122961_4124156 (0 copy vs 2 copy) | 0.904 | 0.982 | 0.734 | 1.314 |
| *C8orf12* | CHR_8_11245641_11247049 (0 copy vs 2 copy) | 0.915 | 0.98 | 0.675 | 1.423 |
| *DOCK5* | CHR_8_25066884_25070636 (0 copy vs 2 copy) | 0.81 | 1.036 | 0.774 | 1.388 |
| *ADAM5, ADAM3A* | CHR_8_39233344_39387179 (0 copy vs 1 or 2 copy) | 0.36 | 0.831 | 0.559 | 1.235 |
| *GDAP1* | CHR_8_75364528_75366830 (0 copy vs 2 copy) | 0.966 | 1.007 | 0.747 | 1.357 |
| *RP11-149P24.1* | CHR_8_137160319_137163816 (0 copy vs 2 copy) | 0.091 | 0.742 | 0.525 | 1.049 |
| *TJP2* | CHR_9_71741217_71743100 (0 copy vs 2 copy) | 0.059 | 0.756 | 0.565 | 1.011 |
| *GABBR2* | CHR_9_101309058_101311079 (0 copy vs 2 copy) | 0.756 | 1.057 | 0.745 | 1.499 |
| *VTI1BP4, WDR34* | CHR_9_131412549_131413853 (0 copy vs 2 copy) | 0.882 | 0.977 | 0.719 | 1.327 |
| *VTI1BP4, WDR34* | CHR_9_131412549_131413885 (0 copy vs 2 copy) | 0.183 | 0.822 | 0.616 | 1.097 |
| *RP11-98L5.4* | CHR_9_138479177_138480145 (0 copy vs 2 copy) | 0.805 | 1.038 | 0.772 | 1.396 |
| *LINC00704* | CHR_10_4708627_4710298 (0 copy vs 2 copy) | 0.724 | 1.053 | 0.789 | 1.406 |
| *PDSS1* | CHR_10_27000558_27001814 (0 copy vs 2 copy) | 0.645 | 0.932 | 0.691 | 1.257 |
| *C10orf11* | CHR_10_78255873_78260694 (0 copy vs 2 copy) | 0.9 | 1.02 | 0.752 | 1.384 |
| *MINPP1* | CHR_10_89275888_89276407 (0 copy vs 1 or 2 copy) | 0.943 | 0.989 | 0.738 | 1.326 |
| *LGI1* | CHR_10_95545536_95546273 (0 copy vs 2 copy) | 0.879 | 1.04 | 0.63 | 1.715 |
| *GUCY2GP* | CHR_10_114113589_114116575 (0 copy vs 2 copy) | 0.096 | 0.667 | 0.415 | 1.074 |
| *PPAPDC1A* | CHR_10_122226947_122228534 (0 copy vs 2 copy) | 0.428 | 0.842 | 0.551 | 1.288 |
| *TRIM5* | CHR_11_5760106_5762286 (0 copy vs 2 copy) | 0.761 | 1.066 | 0.707 | 1.606 |
| *TMEM41B* | CHR_11_9324025_9324496 (0 copy vs 2 copy) | 0.871 | 0.972 | 0.692 | 1.367 |
| *DNAJC24* | CHR_11_31394060_31397428 (0 copy vs 1 or 2 copy) | 0.935 | 0.983 | 0.644 | 1.498 |
| *RP11-430H10.4* | CHR_11_45430401_45431405 (0 copy vs 2 copy) | 0.581 | 0.914 | 0.663 | 1.259 |
| *PC* | CHR_11_66712229_66713105 (0 copy vs 2 copy) | 0.251 | 1.22 | 0.869 | 1.711 |
| *ETV6* | CHR_12_12026506_12026937 (0 copy vs 2 copy) | 0.679 | 0.903 | 0.555 | 1.468 |
| *SLC15A5* | CHR_12_16420184_16420943 (0 copy vs 2 copy) | 0.777 | 1.056 | 0.725 | 1.538 |
| *RP11-352M15.1* | CHR_12_45903118_45909531 (0 copy vs 1 or 2 copy) | 0.668 | 0.931 | 0.673 | 1.289 |
| *LHFP* | CHR_13_39934551_39935151 (0 copy vs 2 copy) | 0.366 | 0.871 | 0.645 | 1.176 |
| *DLEU1* | CHR_13_51069352_51072600 (0 copy vs 2 copy) | 0.827 | 1.033 | 0.775 | 1.376 |
| *NALCN* | CHR_13_101894125_101896318 (0 copy vs 2 copy) | 0.077 | 0.741 | 0.532 | 1.033 |
| *RP11-624L4.1* | CHR_15_39372623_39373245 (0 copy vs 2 copy) | 0.156 | 1.3 | 0.905 | 1.869 |
| *THSD4* | CHR_15_71881673_71882625 (0 copy vs 2 copy) | 0.772 | 0.936 | 0.597 | 1.466 |
| *SCAPER* | CHR_15_76891342_76895185 (0 copy vs 1 or 2 copy) | 0.759 | 0.949 | 0.681 | 1.323 |
| *RP11-661P17.1* | CHR_15_91981864_91983360 (0 copy vs 2 copy) | 0.601 | 1.086 | 0.797 | 1.48 |
| *CNOT1* | CHR_16_58647399_58649650 (0 copy vs 2 copy) | 0.409 | 1.148 | 0.827 | 1.594 |
| *CNTNAP4* | CHR_16_76540062_76543447 (0 copy vs 2 copy) | 0.875 | 0.977 | 0.733 | 1.302 |
| *WWOX* | CHR_16_78373700_78384735 (0 copy vs 1 or 2 copy) | 0.777 | 0.953 | 0.681 | 1.333 |
| *NXN* | CHR_17_724239_724598 (0 copy vs 2 copy) | 0.937 | 0.988 | 0.732 | 1.334 |
| *ACACA* | CHR_17_35755867_35758648 (0 copy vs 1 or 2 copy) | 0.16 | 0.74 | 0.487 | 1.126 |
| *MSI2* | CHR_17_55688120_55689796 (0 copy vs 2 copy) | 0.857 | 0.973 | 0.72 | 1.315 |
| *AQP4-AS1, CHST9* | CHR_18_24571673_24572190 (0 copy vs 2 copy) | 0.74 | 0.935 | 0.629 | 1.391 |
| *MYO5B* | CHR_18_47695103_47698268 (0 copy vs 2 copy) | 0.2 | 1.323 | 0.862 | 2.031 |
| *RP11-176N18.2* | CHR_18_75267039_75267968 (0 copy vs 2 copy) | 0.517 | 0.902 | 0.661 | 1.232 |
| *ZNF57* | CHR_19_2909643_2910369 (0 copy vs 2 copy) | 0.503 | 1.135 | 0.783 | 1.645 |
| *ZNF490* | CHR_19_12694963_12697389 (0 copy vs 2 copy) | 0.82 | 1.034 | 0.776 | 1.377 |
| *CHODL* | CHR_21_19327135_19328810 (0 copy vs 1 or 2 copy) | 0.621 | 1.125 | 0.705 | 1.796 |
| *RPL31P1, HSF2BP* | CHR_21_44970373_44973184 (0 copy vs 2 copy) | 0.824 | 0.965 | 0.706 | 1.32 |
| *SLC25A18* | CHR_22_18058001_18059664 (0 copy vs 2 copy) | 0.876 | 0.972 | 0.682 | 1.387 |
| *AP000351.9* | CHR_22_24365041_24367511 (0 copy vs 2 copy) | 0.338 | 0.81 | 0.526 | 1.247 |
| *RNU7-167P* | CHR_22_35645524_35646052 (0 copy vs 2 copy) | 0.085 | 0.774 | 0.577 | 1.036 |

CHR: Chromosome; CI: confidence interval; HR: hazards ratio; PH: proportional hazards. Models are adjusted for stage, location, and MSI status. P-values less than 0.05 are shown in bold fonts.

**Supporting Information 4 - Table 2.** Results of the age-stratified Multivariable Cox regression analysis for the INDELs/CNVs

| **Gene** | **INDEL/CNV** | **p-value** | **HR** | **95% CI (lower)** | **95% CI (higher)** | ***p-value for PH** |
| --- | --- | --- | --- | --- | --- | --- |
| *PADI4* | CHR_1_17676291_17677196 (0 CN vs 2 CN) | 0.7079 | 1.0935 | 0.685 | 1.7455 | 0.8309 |
| *DAB1* | CHR_1_58744143_58744663 (0 CN vs 2 CN) | 0.9091 | 0.9804 | 0.698 | 1.3771 | 0.5063 |
| *RPL31P12* | CHR_1_72766413_72811692 (0 CN vs 2 CN) | 0.9304 | 0.9865 | 0.7268 | 1.3389 | 0.7636 |
| *GBP3* | CHR_1_89476427_89478432 (0 CN vs 2 CN) | 0.9685 | 1.0094 | 0.6351 | 1.6042 | 0.925 |
| *TGFBR3* | CHR_1_92232111_92233227 (0 CN vs 2 CN) | **0.0454** | 0.5211 | 0.2752 | 0.9867 | 0.2979 |
| *LCE3C, LCE3B* | CHR_1_152556085_152586939 (0 CN vs 2 or 3 CN) | 0.8769 | 1.0257 | 0.7438 | 1.4144 | 0.2937 |
| *NME7* | CHR_1_169207360_169241309 (0 CN vs 1 or 2 CN) | 0.96 | 1.0123 | 0.627 | 1.6344 | **0.0413** |
| *TDRD5* | CHR_1_179607382_179607936 (0 CN vs 2 CN) | 0.7219 | 1.0799 | 0.7072 | 1.6491 | 0.0553 |
| *C4BPA* | CHR_1_207292578_207293178 (0 CN vs 2 CN) | 0.6666 | 1.0652 | 0.7992 | 1.4197 | 0.0741 |
| *LTBP1* | CHR_2_33224605_33227187 (0 CN vs 2 CN) | 0.7589 | 1.0756 | 0.6755 | 1.7125 | 0.7016 |
| *AC073218.1* | CHR_2_34698447_34736476 (0 CN vs 1 or 2 CN) | 0.1774 | 1.302 | 0.8873 | 1.9105 | 0.4287 |
| *C2orf73* | CHR_2_54565729_54567441 (0 CN vs 2 CN) | 0.658 | 0.9334 | 0.6881 | 1.2663 | 0.3946 |
| *C2orf73* | CHR_2_54565729_54567590 (0 CN vs 1 or 2 CN) | 0.6066 | 0.9161 | 0.6562 | 1.2788 | 0.8349 |
| *SNRNP27, MXD1* | CHR_2_70125092_70125504 (0 CN vs 2 CN) | 0.3241 | 0.8092 | 0.5313 | 1.2325 | 0.7296 |
| *REV1* | CHR_2_100103752_100105013 (0 CN vs 2 CN) | 0.2135 | 1.2473 | 0.8806 | 1.7666 | 0.7126 |
| *TANC1* | CHR_2_159959587_159961014 (0 CN vs 2 CN) | 0.2936 | 0.8409 | 0.6086 | 1.1619 | 0.4102 |
| *TANC1* | CHR_2_159959587_159961451 (0 CN vs 2 CN) | 0.6495 | 0.9113 | 0.6106 | 1.3602 | 0.2303 |
| *PPP1R1C* | CHR_2_182856938_182857477 (0 CN vs 2 CN) | 0.4582 | 1.1819 | 0.7601 | 1.8377 | 0.4066 |
| *AC072062.1* | CHR_2_215728845_215730688 (0 CN vs 2 CN) | 0.9146 | 0.9767 | 0.6346 | 1.5031 | 0.6646 |
| *NIFKP7, OSBPL10* | CHR_3_32102055_32106725 (0 CN vs 1 or 2 CN) | 0.5554 | 1.1116 | 0.7821 | 1.5798 | 0.6074 |
| *SCAP* | CHR_3_47490712_47493338 (0 CN vs 2 CN) | 0.2445 | 0.7824 | 0.5175 | 1.1828 | 0.0841 |
| *CMSS1, FILIP1L* | CHR_3_99628822_99629567 (0 CN vs 1 or 2 CN) | **0.015** | 1.6936 | 1.1076 | 2.5896 | 0.3444 |
| *LINC00883, RP11-446H18.5* | CHR_3_107038162_107040253 (0 CN vs 2 CN) | 0.8988 | 0.9707 | 0.6133 | 1.5362 | 0.8449 |
| *SLC12A8* | CHR_3_124936371_124936911 (0 CN vs 1 or 2 CN) | 0.8107 | 1.0467 | 0.7204 | 1.5208 | 0.573 |
| *CPNE4* | CHR_3_131708352_131713017 (0 CN vs 1 or 2 CN) | 0.1825 | 1.3172 | 0.8785 | 1.9751 | 0.7708 |
| *PCCB* | CHR_3_136021052_136026101 (0 CN vs 1 or 2 CN) | 0.7009 | 1.0883 | 0.7067 | 1.6761 | 0.1133 |
| *IQCJ-SCHIP1* | CHR_3_159257057_159257610 (0 CN vs 2 CN) | 0.4461 | 1.191 | 0.7597 | 1.8673 | 0.6704 |
| *RP11-10O22.1* | CHR_3_162765807_162769007 (0 CN vs 2 CN) | 0.6661 | 0.9383 | 0.7025 | 1.2531 | 0.287 |
| *LEPREL1* | CHR_3_189737354_189740440 (0 CN vs 1 or 2 CN) | 0.1296 | 1.3983 | 0.9064 | 2.1569 | 0.8318 |
| *RP11-143P4.2* | CHR_3_192875738_192885153 (0 CN vs 2 or 4 CN) | **0.0394** | 1.3586 | 1.0149 | 1.8186 | 0.9002 |
| *CCSER1* | CHR_4_91933043_91935779 (0 CN vs 2 CN) | 0.7505 | 0.9307 | 0.5981 | 1.4485 | 0.125 |
| *NDST4* | CHR_4_115928747_115929279 (0 CN vs 2 CN) | 0.1438 | 0.6551 | 0.3715 | 1.1551 | 0.8987 |
| *LINC00616* | CHR_4_138966505_138967151 (0 CN vs 2 CN) | 0.9368 | 0.9823 | 0.6315 | 1.5279 | 0.9605 |
| *SMAD1* | CHR_4_146438871_146439991 (0 CN vs 1 or 2 CN) | 0.8007 | 1.0528 | 0.7062 | 1.5695 | 0.469 |
| *TMEM192* | CHR_4_166003471_166004657 (0 CN vs 2 CN) | 0.3689 | 1.1755 | 0.8261 | 1.6727 | 0.548 |
| *GALNTL6* | CHR_4_172989075_172992876 (0 CN vs 2 CN) | 0.823 | 1.0397 | 0.7393 | 1.462 | 0.733 |
| *LINC00290* | CHR_4_182056607_182057107 (0 CN vs 2 CN) | 0.8276 | 1.0352 | 0.7583 | 1.4131 | 0.7641 |
| *PDLIM3* | CHR_4_186441932_186444023 (0 CN vs 2 CN) | 0.1215 | 0.7969 | 0.5978 | 1.0622 | 0.1884 |
| *PDLIM3* | CHR_4_186441932_186444110 (0 CN vs 2 CN) | 0.2439 | 1.2318 | 0.8674 | 1.7493 | **0.0119** |
| *FAM149A* | CHR_4_187093557_187098071 (0 CN vs 2 CN) | 0.7014 | 1.0633 | 0.7771 | 1.4548 | 0.2327 |
| *CTD-3080P12.3* | CHR_5_1178511_1180425 (0 CN vs 2 CN) | 0.2506 | 1.2269 | 0.8655 | 1.7391 | 0.9494 |
| *GUSBP1* | CHR_5_21450792_21452439 (0 CN vs 2 CN) | 0.7988 | 0.9479 | 0.6284 | 1.4299 | **0.0218** |
| *CTC-254B4.1* | CHR_5_106324802_106326299 (0 CN vs 2 CN) | 0.9655 | 0.9919 | 0.6863 | 1.4336 | 0.2209 |
| *SPINK14* | CHR_5_147553186_147554186 (0 CN vs 2 CN) | 0.6513 | 0.9316 | 0.6851 | 1.2668 | 0.5383 |
| *EXOC2* | CHR_6_666535_667756 (0 CN vs 2 CN) | 0.7092 | 0.945 | 0.7019 | 1.2722 | 0.3524 |
| *snoU13, RNF144B* | CHR_6_18402172_18402672 (0 CN vs 2 CN) | 0.427 | 1.1253 | 0.841 | 1.5057 | 0.966 |
| *PKHD1* | CHR_6_51736175_51736742 (0 CN vs 1 or 2 CN) | 0.2369 | 1.2143 | 0.8803 | 1.675 | 0.3962 |
| *MLIP-AS1, MLIP* | CHR_6_53929777_53933874 (0 CN vs 2 CN) | 0.2985 | 0.8569 | 0.6405 | 1.1465 | 0.9597 |
| *EYS* | CHR_6_65347533_65349159 (0 CN vs 2 CN) | 0.5346 | 0.905 | 0.6606 | 1.24 | 0.6478 |
| *GABRR1* | CHR_6_89921782_89922171 (0 CN vs 2 CN) | 0.2558 | 1.1904 | 0.8813 | 1.6079 | 0.9468 |
| *RP11-517H2.6* | CHR_6_167488211_167489090 (0 CN vs 2 CN) | 0.4816 | 1.1451 | 0.7851 | 1.6701 | 0.8693 |
| *STEAP2-AS1, STEAP2* | CHR_7_89810608_89811996 (0 CN vs 2 CN) | 0.6569 | 0.8966 | 0.554 | 1.4512 | 0.5303 |
| *STEAP2-AS1, STEAP2* | CHR_7_89810608_89812114 (0 CN vs 2 CN) | **0.0372** | 0.5776 | 0.3447 | 0.968 | 0.4002 |
| *AC000370.2* | CHR_7_126048572_126051369 (0 CN vs 2 CN) | 0.3971 | 0.8089 | 0.4951 | 1.3216 | 0.1919 |
| *AC000370.2* | CHR_7_126048572_126051476 (0 CN vs 2 CN) | 0.8387 | 0.961 | 0.6549 | 1.41 | 0.2056 |
| *CNTNAP2* | CHR_7_148074379_148076266 (0 CN vs 2 CN) | 0.4389 | 0.8864 | 0.6531 | 1.203 | 0.7985 |
| *ERICH1* | CHR_8_594761_599201 (0 CN vs 2 CN) | 0.6277 | 0.9268 | 0.6818 | 1.26 | 0.6751 |
| *CSMD1* | CHR_8_4122961_4124156 (0 CN vs 2 CN) | 0.8471 | 0.9717 | 0.7254 | 1.3015 | 0.6223 |
| *C8orf12* | CHR_8_11245641_11247049 (0 CN vs 2 CN) | 0.998 | 1.0005 | 0.6885 | 1.4538 | 0.9594 |
| *DOCK5* | CHR_8_25066884_25070636 (0 CN vs 2 CN) | 0.7665 | 1.0454 | 0.7799 | 1.4011 | 0.9903 |
| *ADAM5, ADAM3A* | CHR_8_39233344_39387179 (0 CN vs 1 or 2 CN) | 0.3411 | 0.8248 | 0.5547 | 1.2263 | 0.8655 |
| *GDAP1* | CHR_8_75364528_75366830 (0 CN vs 2 CN) | 0.8082 | 1.038 | 0.7683 | 1.4022 | 0.1561 |
| *RP11-149P24.1* | CHR_8_137160319_137163816 (0 CN vs 2 CN) | 0.0786 | 0.7328 | 0.5182 | 1.0362 | 0.1977 |
| *TJP2* | CHR_9_71741217_71743100 (0 CN vs 2 CN) | 0.0581 | 0.7543 | 0.5636 | 1.0097 | 0.0958 |
| *GABBR2* | CHR_9_101309058_101311079 (0 CN vs 2 CN) | 0.652 | 1.0839 | 0.7637 | 1.5386 | 0.5429 |
| *VTI1BP4, WDR34* | CHR_9_131412549_131413853 (0 CN vs 2 CN) | 0.9026 | 0.9811 | 0.7223 | 1.3325 | 0.7366 |
| *VTI1BP4, WDR34* | CHR_9_131412549_131413885 (0 CN vs 2 CN) | 0.1921 | 0.8249 | 0.6178 | 1.1015 | 0.866 |
| *RP11-98L5.4* | CHR_9_138479177_138480145 (0 CN vs 2 CN) | 0.8617 | 1.0268 | 0.7627 | 1.3823 | 0.9731 |
| *LINC00704* | CHR_10_4708627_4710298 (0 CN vs 2 CN) | 0.7502 | 1.0482 | 0.7847 | 1.4001 | 0.7738 |
| *PDSS1* | CHR_10_27000558_27001814 (0 CN vs 2 CN) | 0.5513 | 0.9123 | 0.6745 | 1.2339 | 0.4973 |
| *C10orf11* | CHR_10_78255873_78260694 (0 CN vs 2 CN) | 0.757 | 1.0496 | 0.7726 | 1.4258 | 0.5459 |
| *MINPP1* | CHR_10_89275888_89276407 (0 CN vs 1 or 2 CN) | 0.896 | 1.0198 | 0.7604 | 1.3675 | 0.7792 |
| *LGI1* | CHR_10_95545536_95546273 (0 CN vs 2 CN) | 0.9451 | 1.0177 | 0.6168 | 1.6794 | 0.9142 |
| *GUCY2GP* | CHR_10_114113589_114116575 (0 CN vs 2 CN) | 0.0874 | 0.6595 | 0.4091 | 1.063 | 0.1796 |
| *PPAPDC1A* | CHR_10_122226947_122228534 (0 CN vs 2 CN) | 0.3876 | 0.8295 | 0.5429 | 1.2676 | 0.2956 |
| *TRIM5* | CHR_11_5760106_5762286 (0 CN vs 2 CN) | 0.9335 | 1.0177 | 0.6746 | 1.5351 | 0.8085 |
| *TMEM41B* | CHR_11_9324025_9324496 (0 CN vs 2 CN) | 0.9633 | 0.992 | 0.7056 | 1.3947 | 0.8368 |
| *DNAJC24* | CHR_11_31394060_31397428 (0 CN vs 1 or 2 CN) | 0.9041 | 0.9744 | 0.6385 | 1.4869 | 0.3131 |
| *RP11-430H10.4* | CHR_11_45430401_45431405 (0 CN vs 2 CN) | 0.5584 | 0.9086 | 0.6592 | 1.2525 | 0.0612 |
| *PC* | CHR_11_66712229_66713105 (0 CN vs 2 CN) | 0.2875 | 1.2015 | 0.8566 | 1.6852 | 0.7789 |
| *ETV6* | CHR_12_12026506_12026937 (0 CN vs 2 CN) | 0.9327 | 0.9792 | 0.6007 | 1.5962 | 0.5481 |
| *SLC15A5* | CHR_12_16420184_16420943 (0 CN vs 2 CN) | 0.7365 | 1.0668 | 0.7319 | 1.5551 | 0.4241 |
| *RP11-352M15.1* | CHR_12_45903118_45909531 (0 CN vs 1 or 2 CN) | 0.6838 | 0.9347 | 0.6754 | 1.2936 | 0.7158 |
| *LHFP* | CHR_13_39934551_39935151 (0 CN vs 2 CN) | 0.3578 | 0.8681 | 0.6422 | 1.1735 | 0.3508 |
| *DLEU1* | CHR_13_51069352_51072600 (0 CN vs 2 CN) | 0.8121 | 1.0354 | 0.777 | 1.3798 | 0.4998 |
| *NALCN* | CHR_13_101894125_101896318 (0 CN vs 2 CN) | 0.0798 | 0.7427 | 0.5324 | 1.0359 | 0.2969 |
| *RP11-624L4.1* | CHR_15_39372623_39373245 (0 CN vs 2 CN) | 0.2274 | 1.2515 | 0.8694 | 1.8015 | 0.7193 |
| *THSD4* | CHR_15_71881673_71882625 (0 CN vs 2 CN) | 0.7324 | 0.9243 | 0.5885 | 1.4516 | 0.0673 |
| *SCAPER* | CHR_15_76891342_76895185 (0 CN vs 1 or 2 CN) | 0.7548 | 0.9483 | 0.6795 | 1.3235 | 0.956 |
| *RP11-661P17.1* | CHR_15_91981864_91983360 (0 CN vs 2 CN) | 0.5408 | 1.1017 | 0.8077 | 1.5026 | 0.2388 |
| *CNOT1* | CHR_16_58647399_58649650 (0 CN vs 2 CN) | 0.3685 | 1.163 | 0.8369 | 1.616 | 0.1922 |
| *CNTNAP4* | CHR_16_76540062_76543447 (0 CN vs 2 CN) | 0.7307 | 0.9505 | 0.7118 | 1.2692 | 0.4307 |
| *WWOX* | CHR_16_78373700_78384735 (0 CN vs 1 or 2 CN) | 0.9201 | 0.9829 | 0.701 | 1.378 | 0.8013 |
| *NXN* | CHR_17_724239_724598 (0 CN vs 2 CN) | 0.8559 | 0.9726 | 0.7207 | 1.3126 | 0.8732 |
| *ACACA* | CHR_17_35755867_35758648 (0 CN vs 1 or 2 CN) | 0.1951 | 0.7572 | 0.4971 | 1.1534 | 0.1903 |
| *MSI2* | CHR_17_55688120_55689796 (0 CN vs 2 CN) | 0.8083 | 0.9634 | 0.7128 | 1.302 | 0.7803 |
| *AQP4-AS1, CHST9* | CHR_18_24571673_24572190 (0 CN vs 2 CN) | 0.6977 | 0.9242 | 0.621 | 1.3756 | 0.1238 |
| *MYO5B* | CHR_18_47695103_47698268 (0 CN vs 2 CN) | 0.2691 | 1.2759 | 0.8282 | 1.9655 | 0.3915 |
| *RP11-176N18.2* | CHR_18_75267039_75267968 (0 CN vs 2 CN) | 0.4543 | 0.8876 | 0.6494 | 1.2131 | 0.8128 |
| *ZNF57* | CHR_19_2909643_2910369 (0 CN vs 2 CN) | 0.5232 | 1.1288 | 0.7782 | 1.6375 | 0.4248 |
| *ZNF490* | CHR_19_12694963_12697389 (0 CN vs 2 CN) | 0.8367 | 1.0307 | 0.7732 | 1.3739 | 0.2057 |
| *CHODL* | CHR_21_19327135_19328810 (0 CN vs 1 or 2 CN) | 0.5629 | 1.1483 | 0.7187 | 1.8347 | 0.5398 |
| *RPL31P1, HSF2BP* | CHR_21_44970373_44973184 (0 CN vs 2 CN) | 0.8447 | 0.9691 | 0.7082 | 1.3263 | 0.6559 |
| *SLC25A18* | CHR_22_18058001_18059664 (0 CN vs 2 CN) | 0.8535 | 0.967 | 0.6776 | 1.3801 | 0.4156 |
| *AP000351.9* | CHR_22_24365041_24367511 (0 CN vs 2 CN) | 0.2914 | 0.7927 | 0.5148 | 1.2205 | 0.1053 |
| *RNU7-167P* | CHR_22_35645524_35646052 (0 CN vs 2 CN) | 0.0626 | 0.7569 | 0.5646 | 1.0148 | 0.2091 |

CHR: Chromosome; CI: confidence interval; HR: hazards ratio; PH: proportional hazards. Models are stratified for age and adjusted for stage, location, and MSI status. *p-value by the Score test (1). P-values less than 0.05 are shown in bold fonts.

Variants that deviated from the PH assumption are shown with green highlights; these results are only shown for the purpose of the comparison of the results obtained with or without the time-varying coefficients, and the results considering the time-varying coefficients shown in the manuscript should be considered more accurate.

**References**

1. Grambsch P,M, Therneau TM. Proportional hazards tests and diagnostics, based on weighted residuals. Biometrika 1994;81:515-526.
