# Supplementary material for: Germline INDELs and CNVs in a cohort of colorectal cancer patients: their characteristics, associations with relapse‐free survival time, and potential time‐varying effects on the risk of relapse
Source: Cancer Med. 2017 May 23;6(6):1220–32. doi: 10.1002/cam4.1074 (PMC5463068; doi:10.1002/cam4.1074)
Supplement: Supplementary file 5 — Data S5. Variants unique to FCCX cases. [file CAM4-6-1220-s005.docx]

**Supporting Information 5**

**Variants unique to FCCX cases.**

**Supporting Information 5 - Table 1.** INDELs/CNVs that are unique to FCCX cases.

| **CHR** | **START position** | **END position** | ***CHR band** | **CN** | **Length** | **ENSEMBL ID** | **Gene** |
| --- | --- | --- | --- | --- | --- | --- | --- |
| 1 | 104100911 | 104136650 | 1p21.1 | 3 | 35739 | ENSG00000236085, ENSG00000240038 | *ACTG1P4, AMY2B* |
| 1 | 108858527 | 108888654 | 1p13.3 | 3 | 30127 | ENSG00000241361 | *SLC25A24P1* |
| 2 | 1527274 | 1537864 | 2p25.3 | 3 | 10590 | ENSG00000115705 | *TPO* |
| 2 | 24602959 | 24611360 | 2p23.3 | 0 | 8401 | - | *-* |
| 2 | 34698447 | 34729435 | 2p22.3 | 0 | 30988 | ENSG00000226785 | *AC073218.1* |
| 2 | 90125210 | 90283546 | 2p11.2 | 3 | 158336 | ENSG00000178894, ENSG00000254292, ENSG00000253906, ENSG00000241244, ENSG00000224041, ENSG00000211630, ENSG00000240834, ENSG00000211632, ENSG00000211633, ENSG00000242580, ENSG00000239819, ENSG00000235896 | *AC073416.1, IGKV2D-14, IGKV2D-10, IGKV1D-16, IGKV3D-15, IGKV1D-13, IGKV1D-12, IGKV3D-11, IGKV1D-42, IGKV1D-43, IGKV1D-8, IGKV3D-7* |
| 2 | 105662069 | 105665808 | 2q12.1 | 0 | 3739 | ENSG00000135972 | *MRPS9* |
| 3 | 131708352 | 131712742 | 3q22.1 | 1 | 4390 | ENSG00000196353 | *CPNE4* |
| 3 | 162509817 | 162656280 | 3q22.1 | 4 | 146463 | - | *-* |
| 3 | 195453991 | 195472740 | 3q26.1 | 3 | 18749 | ENSG00000242086, ENSG00000176945 | *LINC00969, MUC20* |
| 4 | 10272429 | 10274279 | 4p16.1 | 1 | 1850 | - | *-* |
| 6 | 29862114 | 29890271 | 6p22.1 | 1 | 28157 | ENSG00000231130, ENSG00000233677, ENSG00000235963 | *HLA-T,DDX39BP1, MCCD1P1* |
| 6 | 29879173 | 29905193 | 6p22.1 | 3 | 26020 | ENSG00000227262, ENSG00000230795, ENSG00000228078 | *HCG4B, HLA-K, HLA-U* |
| 7 | 87669005 | 87672670 | 7q21.12 | 1 | 3665 | ENSG00000008277 | *ADAM22* |
| 8 | 11924124 | 12010237 | 8p23.1 | 3 | 86113 | ENSG00000252029, ENSG00000255544, ENSG00000255052, ENSG00000254923, ENSG00000226430, ENSG00000254866, ENSG00000233050, ENSG00000215343, ENSG00000223443 | *RNA5SP253, DEFB108P3, FAM66D, RP11-1236K1.8, USP17L7, DEFB109P3, DEFB130, ZNF705D, USP17L2* |
| 8 | 12232330 | 12251955 | 8p23.1 | 3 | 19625 | ENSG00000227888, ENSG00000254423, ENSG00000255556, ENSG00000242296 | *FAM66A, RP11-351I21.7, RP11-351I21.6, DEFB109P1* |
| 9 | 75800805 | 75808530 | 9q21.13 | 1 | 7725 | - | *-* |
| 10 | 89007613 | 89108950 | 10q23.2 | 1 | 101337 | ENSG00000223482, ENSG00000224914 | *NUTM2A-AS1, LINC00863* |
| 11 | 93683453 | 93688134 | 11q21 | 1 | 4681 | - | *-* |
| 12 | 7997547 | 8116068 | 12p13.31 | 3 | 118521 | ENSG00000173262, ENSG00000059804, ENSG00000222978, ENSG00000201663, ENSG00000241828, ENSG00000255885, ENSG00000176654, ENSG00000255356 | *SLC2A14, SLC2A3, Y_RNA, Y_RNA, RP11-277J24.1, RP11-815D16.1, NANOGP1, RP11-277E18.2* |
| 13 | 57787187 | 57788023 | 13q21.1 | 0 | 836 | - | *-* |
| 15 | 22521113 | 22560308 | 15q11.2 | 1 | 39195 | ENSG00000259098 | *RP11-603B24.2* |
| 15 | 24497572 | 24694058 | 15q11.2 | 3 | 196486 | ENSG00000261621, ENSG00000261598, ENSG00000260760 | *RP11-580I1.2, RP11-107D24.2, PWRN3* |
| 15 | 56790539 | 56811204 | 15q21.3 | 1 | 20665 | - | *-* |
| 16 | 33434576 | 33632860 | 16p11.2 | 3 | 198284 | ENSG00000260518, ENSG00000261580, ENSG00000260308, ENSG00000261153, ENSG00000270467, ENSG00000271178 | *BMS1P8, ENPP7P13, RP11-104C4.4, RP11-104C4.2, IGHV3OR16-12, IGHV3OR16-13* |
| 16 | 78876980 | 78877618 | 16q23.1 | 0 | 638 | ENSG00000186153 | *WWOX* |
| 17 | 44249096 | 44283571 | 17q21.31 | 3 | 34475 | ENSG00000120071, ENSG00000214401 | *KANSL1, KANSL1-AS1* |
| 18 | 61840388 | 61982829 | 18q22.1 | 3 | 142441 | ENSG00000267134, ENSG00000266952 | *RP11-146N18.1, RP11-909B2.1* |

CHR: chromosome; CN: copy number state. *Based on UCSC genome browser (hg19) (1)

**Supporting Information 5 - Table 2.** INDELs/CNVs and genes unique to FCCX cases prior to filtering out the variants based on previous studies

| **CHR** | **START** | **END** | ***CHR band** | **CN** | **Length** | **ENSEMBL ID** | **Gene** |
| --- | --- | --- | --- | --- | --- | --- | --- |
| 1 | 2576908 | 2785671 | 1p36.32 | 0 | 208763 | ENSG00000215912, ENSG00000233234, ENSG00000231630 | TTC34, RP11-740P5.2, RP11-740P5.3 |
| 1 | 63041800 | 63114560 | 1p31.3 | 3 | 72760 | ENSG00000213703, ENSG00000116641, ENSG00000132855, ENSG00000269624 | RP5-849H19.2, DOCK7, ANGPTL3, AL138847.1 |
| 1 | 92234553 | 92642484 | 1p22.1 | 3 | 407931 | ENSG00000239794, ENSG00000266532, ENSG00000233228, ENSG00000230667, ENSG00000224678, ENSG00000233401, ENSG00000069702, ENSG00000137948, ENSG00000172031, ENSG00000189195, ENSG00000069712 | RN7SL653P, RN7SL235P, LPCAT2BP, SETSIP, GAPDHP46, PRKAR1AP, TGFBR3, BRDT, EPHX4, BTBD8, KIAA1107 |
| 2 | 44742517 | 44759941 | 2p21 | 1 | 17424 | ENSG00000143919 | CAMKMT |
| 2 | 182030229 | 182169888 | 2q31.3 | 1 | 139659 | ENSG00000234663 | AC104820.2 |
| 2 | 228575393 | 228607356 | 2q36.3 | 3 | 31963 | ENSG00000135917 | SLC19A3 |
| 3 | 5419668 | 5470493 | 3p26.1 | 1 | 50825 | - | - |
| 3 | 82863216 | 82873921 | 3p12.2 | 1 | 10705 | - | - |
| 4 | 1077575 | 1083914 | 4p16.3 | 3 | 6339 | ENSG00000178222 | RNF212 |
| 4 | 9966771 | 9974186 | 4p16.1 | 3 | 7415 | ENSG00000109667 | SLC2A9 |
| 4 | 80357377 | 80362679 | 4q21.21 | 1 | 5302 | - | - |
| 4 | 86789668 | 86871282 | 4q21.23 | 3 | 81614 | ENSG00000265774, ENSG00000138639 | AC098870.1, ARHGAP24 |
| 4 | 140115744 | 140179888 | 4q31.1 | 3 | 64144 | ENSG00000207384, ENSG00000252362, ENSG00000206722 | Y_RNA, RNU6-506P, RNU6-1074P |
| 4 | 177006484 | 177101060 | 4q34.2 | 3 | 94576 | ENSG00000201516, ENSG00000150627 | SNORA51, WDR17 |
| 5 | 78106062 | 78111731 | 5q14.1 | 1 | 5669 | ENSG00000113273 | ARSB |
| 5 | 128926595 | 129030478 | 5q23.3 | 1 | 103883 | ENSG00000251680, ENSG00000145808 | CTC-575N7.1, ADAMTS19 |
| 6 | 74828682 | 74833405 | 6q13 | 1 | 4723 | ENSG00000223786 | RP11-554D15.1 |
| 6 | 131074746 | 131121992 | 6q23.1 | 1 | 47246 | - | - |
| 6 | 142997132 | 143118715 | 6q24.2 | 3 | 121583 | ENSG00000010818, ENSG00000233138, ENSG00000237851 | HIVEP2, RP1-67K17.3, RP1-67K17.4 |
| 7 | 5883055 | 5919298 | 7p22.1 | 1 | 36243 | ENSG00000235944, ENSG00000265040 | ZNF815P, RN7SL556P |
| 7 | 72169613 | 72255343 | 7q11.22-q11.23 | 3 | 85730 | ENSG00000270694, ENSG00000270555, ENSG00000254184 | RP11-535E8.2, RP11-1394O16.1, TYW1B |
| 11 | 11875747 | 11893217 | 11p15.3 | 1 | 17470 | ENSG00000255492, ENSG00000170242 | CTD-2381F24.1, USP47 |
| 11 | 127748894 | 127761666 | 11q24.2 | 1 | 12772 | - | - |
| 13 | 108041621 | 108055161 | 13q33.3 | 1 | 13540 | ENSG00000204442 | FAM155A |
| 14 | 90390739 | 90470981 | 14q32.11 | 1 | 80242 | ENSG00000259053, ENSG00000140025, ENSG00000042088 | RP11-33N16.3, EFCAB11, TDP1 |
| 15 | 78865893 | 78874073 | 15q25.1 | 3 | 8180 | ENSG00000169684 | CHRNA5 |
| 16 | 12826445 | 12880446 | 16p13.12 | 1 | 54001 | ENSG00000260378, ENSG00000261158, ENSG00000103381 | CTD-2583P5.1, CTD-2583P5.3, CPPED1 |
| 16 | 15066052 | 15221957 | 16p13.11 | 3 | 155905 | ENSG00000261819, ENSG00000238728, ENSG00000260872, ENSG00000260735, ENSG00000188599, ENSG00000270580, ENSG00000250251, ENSG00000179889, ENSG00000157045, ENSG00000085721 | RP11-680G24.4, MIR1972-1, RP11-680G24.5, RP11-72I8.1, NPIPP1, RP11-1186N24.5, PKD1P6, PDXDC1, NTAN1, RRN3 |
| 16 | 16246164 | 16261251 | 16p13.11 | 1 | 15087 | ENSG00000091262 | ABCC6 |
| 18 | 51124626 | 51137738 | 18q21.2 | 1 | 13112 | ENSG00000242945 | RPL29P32 |

CHR: chromosome; CN: copy number state. *Based on UCSC genome browser (hg19) (1)

**References**

1. Kent WJ, Sugnet CW, Furey TS, Roskin KM, Pringle TH, Zahler AM, et al. The human genome browser at UCSC. Genome Res 2002 Jun;12(6):996-1006.
